# Supplementary material for: Rat cell-derived kidney generation via interspecies blastocyst complementation in an Osr1-KO mouse model
Source: Stem Cell Reports. 2026 Jun 11;21(7):102957. doi: 10.1016/j.stemcr.2026.102957 (PMC13385422; doi:10.1016/j.stemcr.2026.102957)
Supplement: Document S1. Figures S1–S6 and Tables S1–S8 [file mmc1.pdf]

Stem Cell Reports, Volume *21*

Supplemental Information

Rat cell-derived kidney generation via interspecies blastocyst complementation in an *Osr1*-KO mouse model

Shunsuke Yuri and Ayako Isotani

Fig.S1

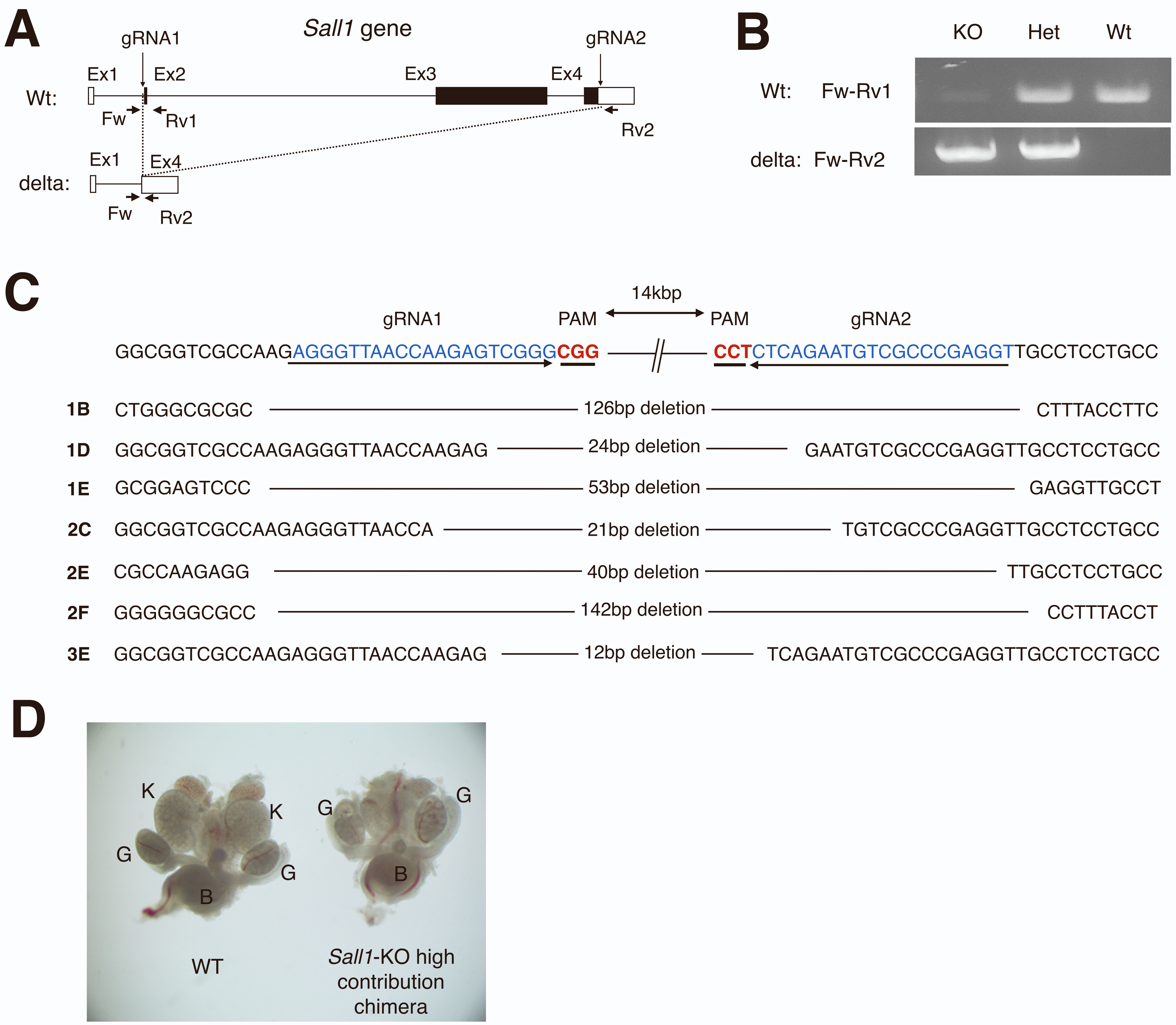

Figure S1. Generation of *Sall1*-KO ESC lines, related to Figure 1.

(A) Strategy for generating the *Sall1* knockout (KO) model. Both gRNA1 and gRNA2 were used to delete exons 1–4 of the *Sall1* gene.

(B) Genotypes of *Sall1*-KO, *Sall1* heterozygous (Het), and *Sall1* WT ESCs.

(C) Mutation patterns of the obtained *Sall1*-KO ESC lines.

(D) Representative images of the urogenital region in WT and *Sall1*-KO chimeras with high *Sall1*-KO cell contribution.

Fig.S2

A

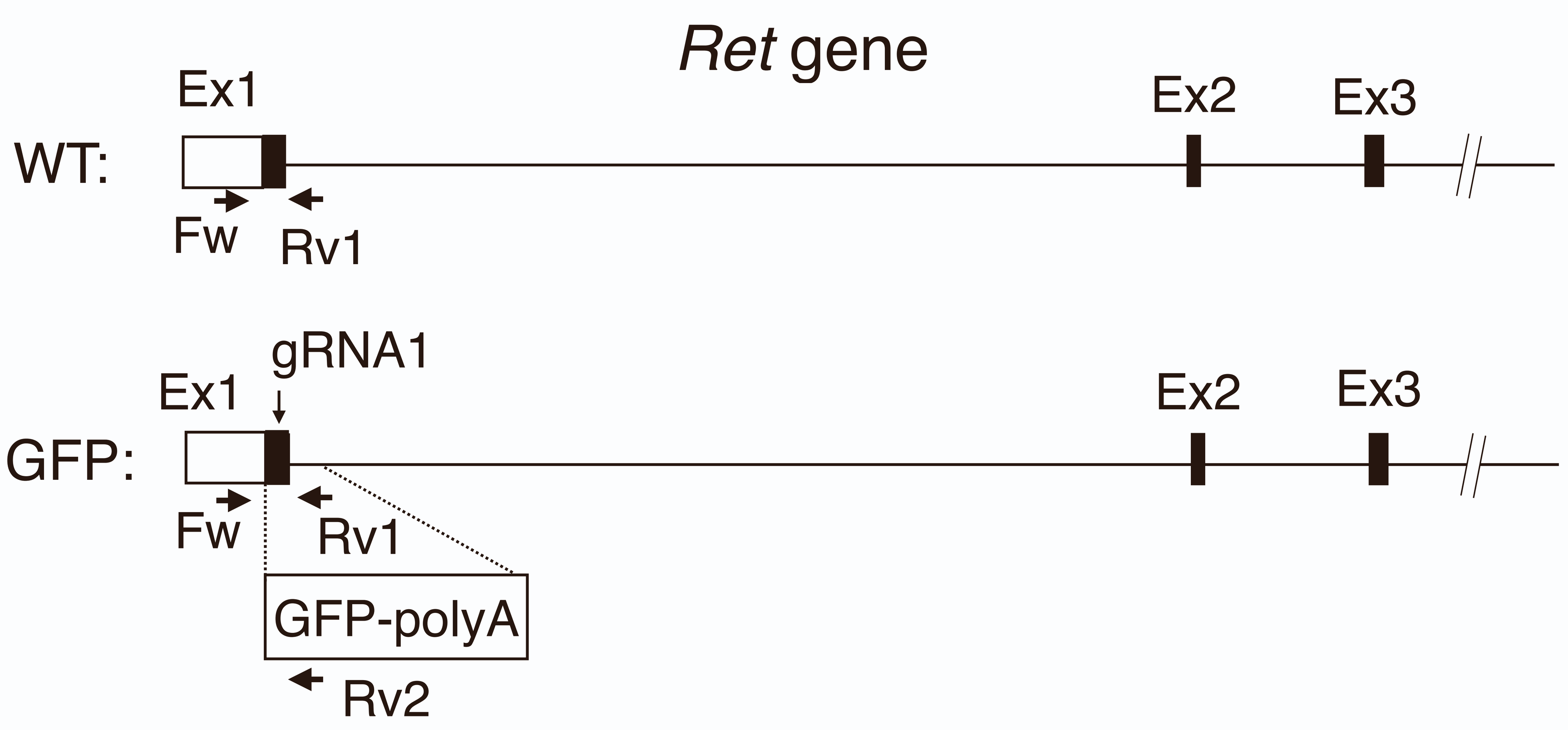

B

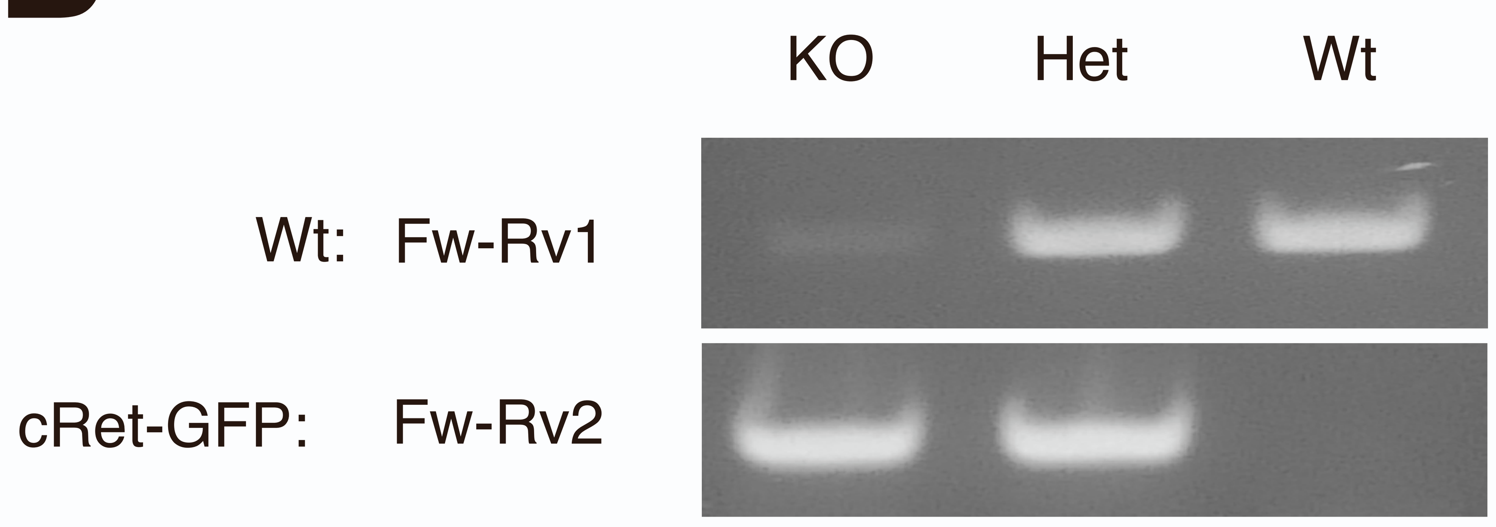

C

clone	allele 1	allele 2
**7B**	GFP insertion	1 bp addition (Frame shift)
**7E**	GFP insertion	1 bp addition (Frame shift)
**9D**	GFP insertion	GFP insertion

D

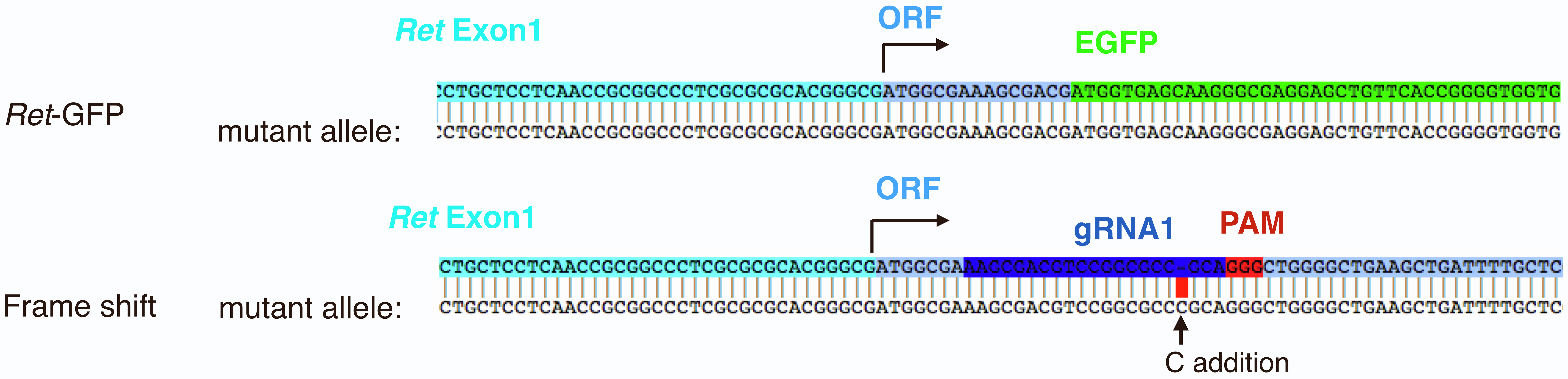

E

clone	*Ret* allele 1	*Ret* allele 2
**1A**	GFP insertion	GFP insertion
**1H**	GFP insertion	GFP insertion
**5D**	GFP insertion	GFP insertion

Figure S2. Generation of *Ret*-KO ESC lines, related to Figure 2.

(A) Strategy for generating the *Ret* knockout (KO) model. A GFP–polyA cassette was inserted in-frame within the *Ret* gene.

(B) Genotypes of *Ret*-KO, *Ret* heterozygous (Het), and *Ret* WT ESCs.

(C–D) Mutation patterns of the obtained *Ret*-KO ESC lines.

(E) Mutation patterns of the obtained *Ret*/*Sall1*-KO ESC lines.

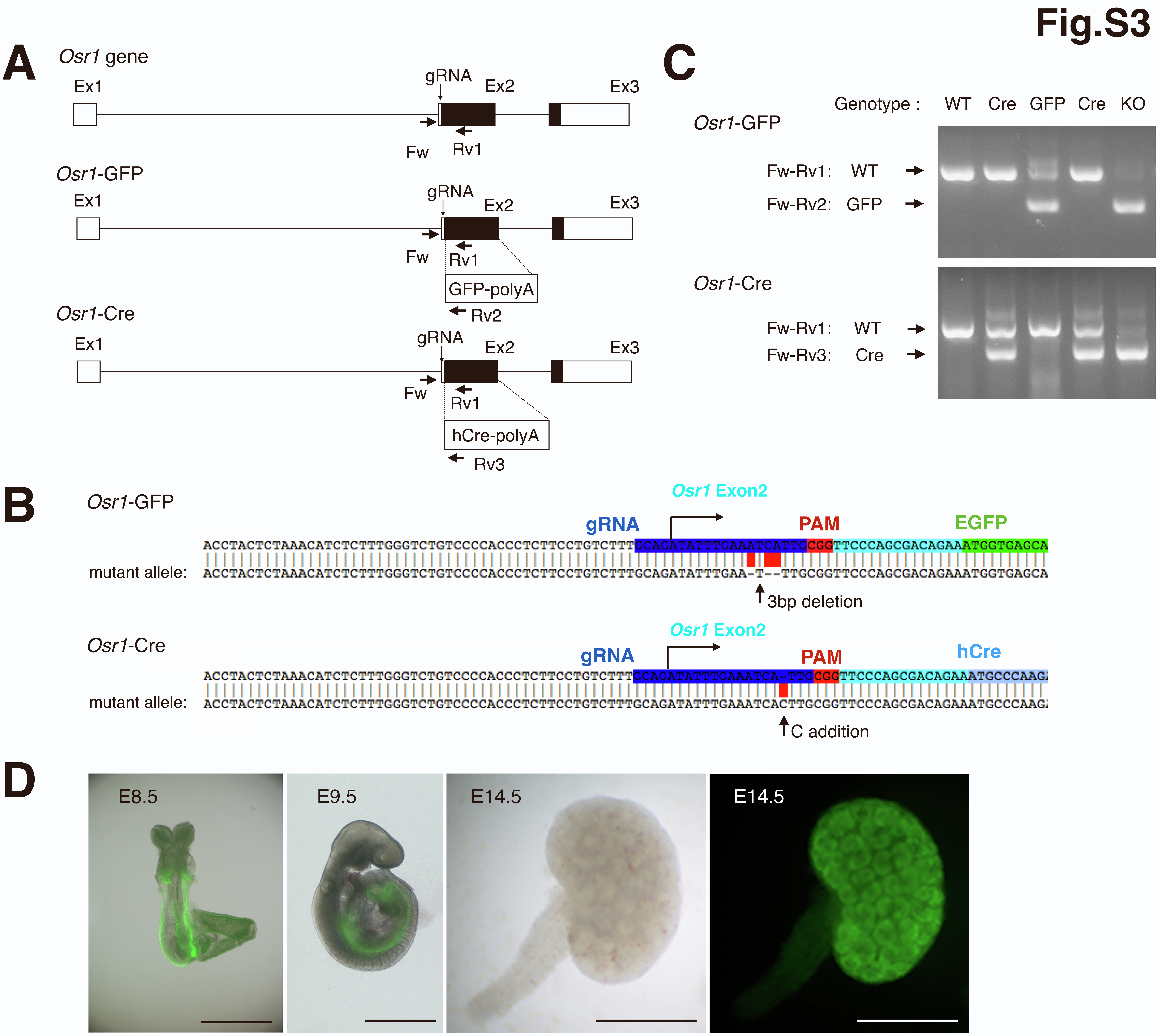

Figure S3. Generation of *Osr1*-GFP and *Osr1*-Cre ESC lines, related to Figure 3 and 4.
(A) Strategy for generating the *Osr1*-GFP and *Osr1*-Cre ESC lines. A GFP–polyA cassette or an hCre–polyA cassette was inserted in-frame within the *Osr1* gene.
(B) Mutation patterns of the obtained *Osr1*-GFP and *Osr1*-Cre ESC lines.
(C) Genotypes of the embryos obtained from *Osr1*-GFP and *Osr1*-Cre mouse lines.
(D) Representative images of GFP expression patterns in *Osr1*-GFP embryos at E8.5 and E9.5, and in the E14.5 kidney. Scale bars: 1 mm (E8.5 and E9.5), 500 µm (E14.5 kidney).

Fig.S4

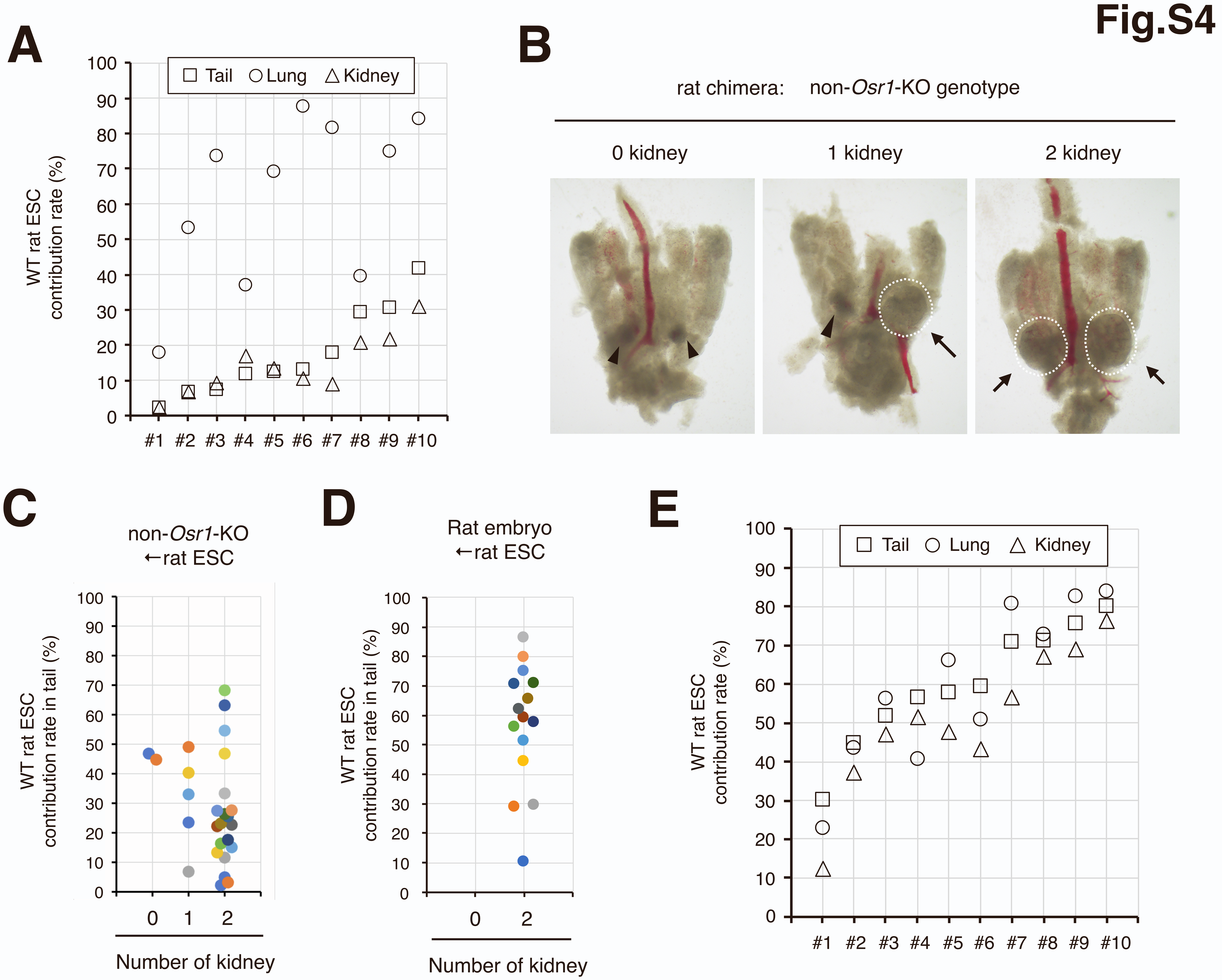

Figure S4. Characterization of kidneys in the rat ESC chimeras, related to Figure 5.

(A) Representative cellular contribution rates of the injected rat WT cells in the tail, lung, and kidney of chimeras with a non-*Osr1*-KO genotype.

(B) Representative urogenital images obtained from chimeras with a non-*Osr1*-KO genotype. The black arrow and white dot circle indicate a kidney, and the black arrowhead indicates a vestigial kidney structure.

(C) Relationship between the cellular contribution rate of donor (rat) cells in the tail and the presence of kidneys in non-*Osr1*-KO-rat chimeras. Non-*Osr1*-KO–rat chimeras without kidneys (n=2), with one kidney (n=5), and with two kidneys (n = 20) were analyzed.

(D) Relationship between the cellular contribution rate of donor (rat) cells in the tail and the presence of kidneys in rat-rat chimeras. WT rat–rat chimeras with two kidneys (n = 15) were analyzed.

(E) Representative cellular contribution rates of the injected rat WT cells in the tail, lung, and kidney of rat–rat chimeras.

Fig.S5

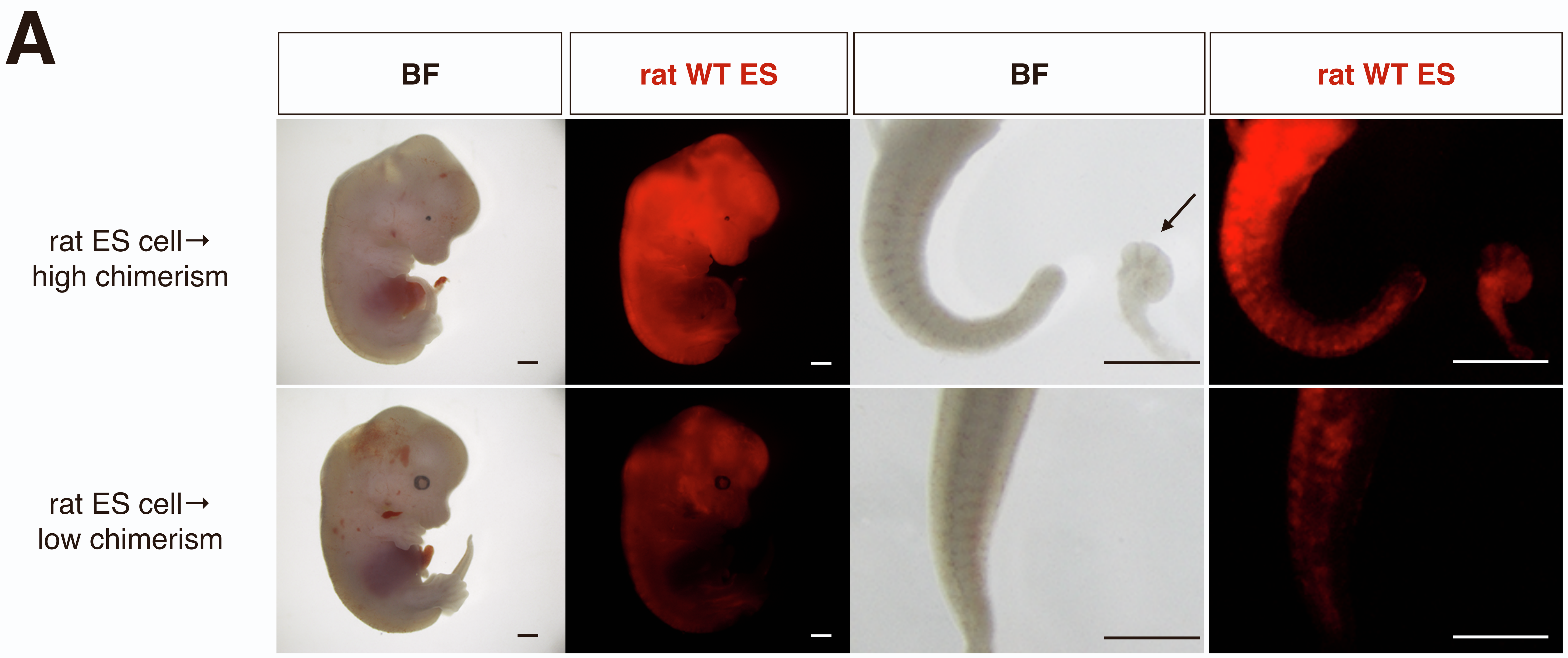

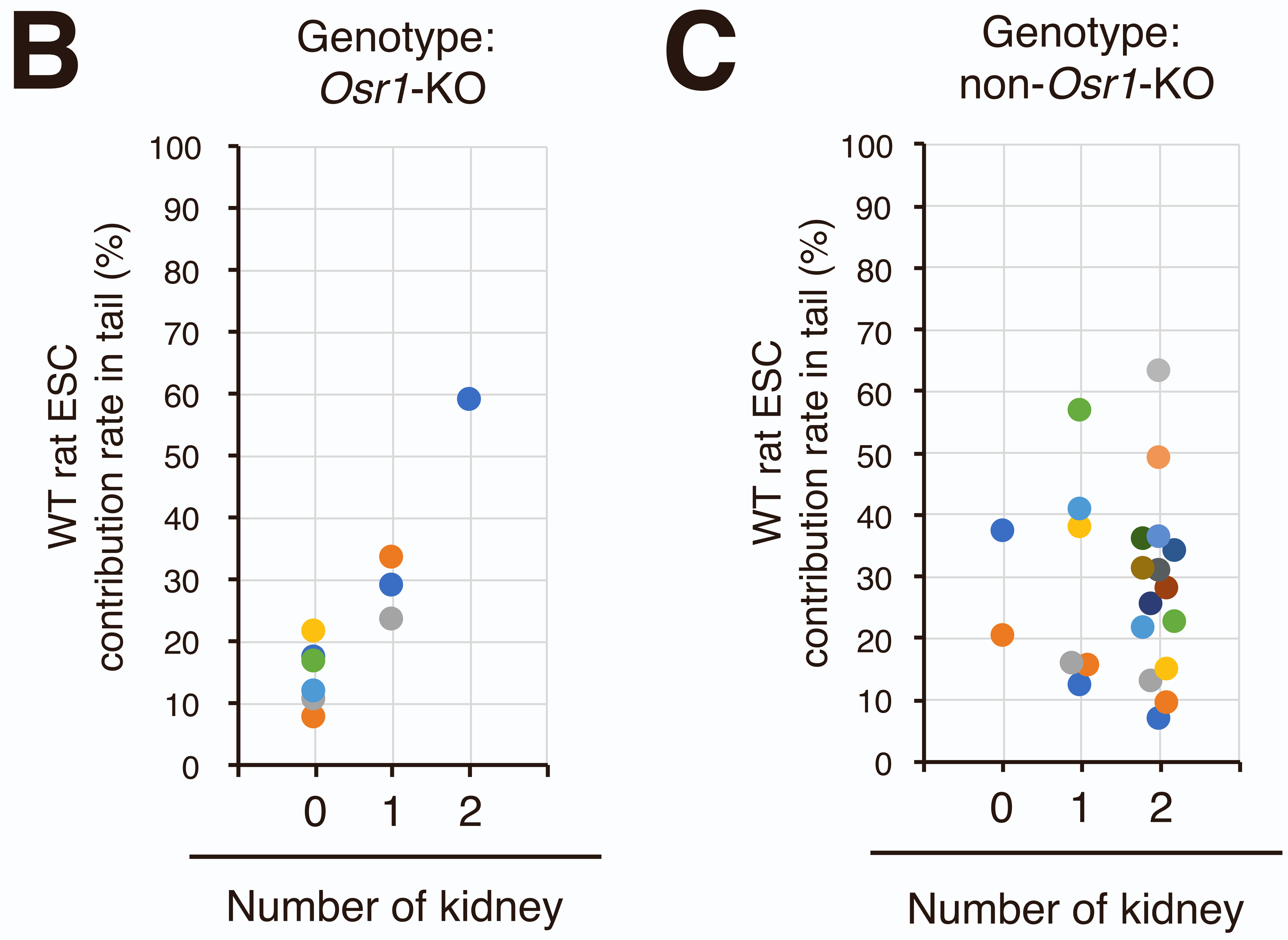

D

Genotype	WT	Het	KO
Chimera	11	25	10
non-Chimera	12	32	8

Figure S5. Interspecies BC method for generating rat kidney with *Osr1*-KO mouse model, related to Figure 5.

(A) Representative images of embryos and kidneys derived from chimeras generated from mouse *Osr1*-KO embryos and rat ESCs (RFP+: rRFP5-2), showing high rat contribution (upper) and low rat contribution (lower). Chimeras with high rat contribution contained kidneys (black arrow) in the *Osr1*-KO background, and RFP-expressing kidneys were observed. Scale bars, 1 mm.

(B) Relationship between the cellular contribution rate of injected rat WT cells in the tail and the presence of kidneys in *Osr1*-KO and WT rat ESC chimeras. *Osr1*-KO-rat WT ESC (rRFP5-2) chimeras without kidneys (n = 6), one kidney (n = 3), or two kidneys (n = 1) were analyzed.

(C) Relationship between the cellular contribution rate of donor (rat) cells in the tail and the presence of kidneys in non-*Osr1*-KO ESC chimeras. Non-*Osr1*-KO-rat ESC (rRFP5-2) chimeras without kidneys (n = 2), with one kidney (n = 6), or with two kidneys (n = 15) were analyzed.

(D) Genotype results of interspecies blastocyst complementation using rRFP5-2.

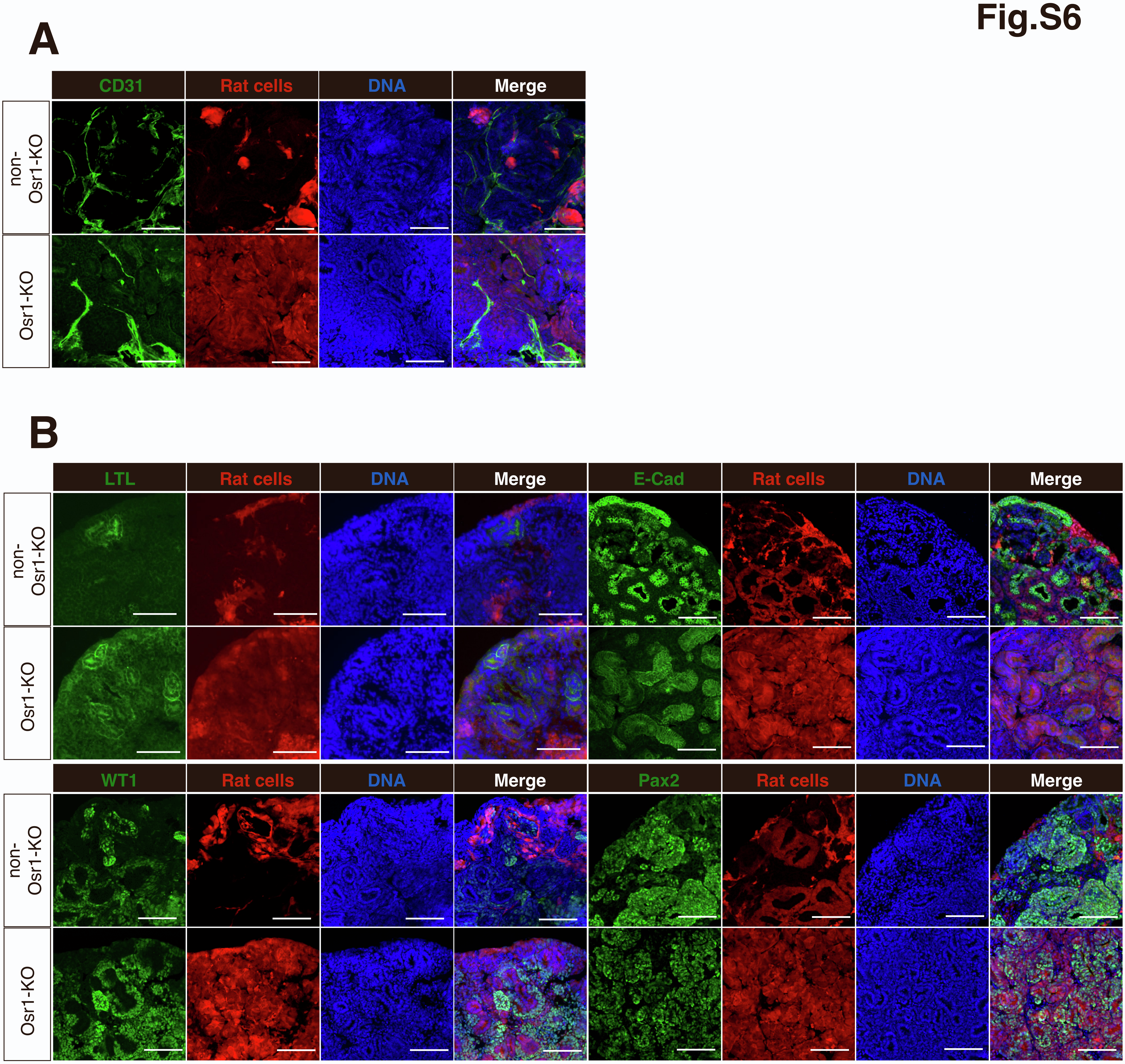

Figure S6. Immunofluorescence analysis of kidneys in the interspecies chimeras, related to Figure 5.

(A) Representative immunostaining image of CD31 in kidney of *Osr1*-KO and rat WT cell chimera or non-*Osr1*-KO and rat WT cell chimera. The kidney tissues were organ-cultured for 5-7 days. Scale bars, 100 µm.

(B) Representative immunostaining image of LTL, E-Cad, WT1 and Pax2 in kidney of *Osr1*-KO and rat WT cell chimera or non-*Osr1*-KO and rat WT cell chimera. The kidney tissues were organ-cultured for 5-7 days. Scale bars, 100 µm.

Table S1. Results of *Sall1*-KO ESC injection with the rBC method (donor: mouse ESCs; host: mouse embryo), related to Figure 1

ESC line	Transplantation	Implantation	Live embryos	RFP+ chimera
Sall1-KO ESCs (1B)	31	19	10	11
Sall1-KO ESCs (1D)	31	22	6	3
Sall1-KO ESCs (1E)	30	23	13	8
Sall1-KO ESCs (2C)	30	22	11	6
Sall1-KO ESCs (2E)	30	22	13	9
Sall1-KO ESCs (2F)	30	23	10	7
Sall1-KO ESCs (3E)	71	40	20	14
WT ESCs (R01-09)	129	85	48	37

Table S2. Results of *Ret*-KO and *Ret/Sall1*-DKO ESC injection with the rBC method (donor: mouse ESCs; host: mouse embryo), related to Figure 2

ESC line	Transplantation	Implantation	Live embryos	RFP+ chimera
Ret-KO ESCs (7B)	40	33	16	7
Ret-KO ESCs (7E)	183	116	81	59
Ret-KO ESCs (9D)	20	18	8	3
Ret/Sall1-DKO ESCs (1A)	63	49	18	9
Ret/Sall1-DKO ESCs (1H)	30	22	6	4
Ret/Sall1-DKO ESCs (5D)	107	67	45	36

Table S3. Results of *Osr1*-KO ESC and WT ESC injection with the rBC method (donor: mouse ESCs; host: mouse embryo), related to Figure 3

ESC line	Transplantation	Implantation	Live embryos	RFP+ chimera
Osr1 -KO ESCs (#4)	15	9	7	7
Osr1 -KO ESCs (#6)	16	9	5	2
Osr1 -KO ESCs (#9)	18	8	7	6
Osr1 -KO ESCs (#12)	45	31	18	13
Osr1 -KO ESCs (#15)	48	29	22	14
Osr1 -KO ESCs (#23)	46	31	27	15
Osr1 -KO ESCs (#24)	42	22	18	11
WT ESCs (#3-2)	20	15	13	12

Table S4. Results of mouse WT ESC injection with the intraspecies BC method (donor: mouse ESCs; host: mouse embryo), related to Figure 4

ESC line	Transplantation	implantation	live embryos	RFP+ chimera
WT mouse ESCs	868	514	318	153

Table S5. Results of rat WT ESC injection with the interspecies BC method (donor: rat ESCs; host: mouse embryo), related to Figure 5 and Figure S5

ESC line	Transplantation	implantation	live embryos	RFP+ chimera
rDby-RFP	724	385	164	60
rRFP-5-2	817	443	98	46

Table S6. Results of rat WT ESC injection in rat embryos (donor: rat ESCs; host: rat embryo), related to Figure 5 and Figure S4

ESC line	Transplantation	RFP+ chimera
rDby-RFP	76	23

Table S7. Oligonucleotide information, related to STAR Methods

ID	oligo name	sequence	comment
#1	Sall1-sgRNA1-F	caccAGGGTTAACCAAGAGTCGGG	
#2	Sall1-sgRNA1-R	aaacCCCGACTCTTGGTTAACCCT	
#3	Sall1-sgRNA2-F	caccACCTCGGGCGACATTCTGAG	
#4	Sall1-sgRNA2-R	aaacCTCAGAATGTCGCCCGAGGT	
#5	Sall1-KO check F	ATTAAGGCGAGCGGGTTTGA	
#6	Sall1-KO check R	AGAGGCATTTGCTCTTGTAAGG	#5 & #6: Sall1-KO allele check
#7	Sall1-WT check R	GAAAGCTCCAGCCGGGATAG	#5 & #7: Sall1-WT allele check
#8	Ret-sgRNA1-F	caccAAGCGACGTCCGGCGCCGCA	
#9	Ret-sgRNA1-R	aaacTGCGGCGCCGGACGTCGCTT	
#10	pLSODN-4D+Ret-Left-F	atgcctgcaggctcttcgatATCCTTCCCAACACCCCTGTAG	
#11	Ret-Left-R	CGTCGCTTTCGCCATCG	#10 & #11: left-arm amplification for Ret-GFP
#12	MluI+Ret Right arm F	ggcgatggcgaaagcgacgacgcgtAGTAGACTGCTCCGCCAAAG	
#13	pLSODN-4D+Ret-Right-R	gtggcaatgcccgggatgatCACCTTAGGACCCAGGAACA	#12 & #13: right-arm amplification for Ret-GFP
#14	Ret-EGFP-F	ggcgatggcgaaagcgacgATGGTGAGCAAGGGCGAG	
#15	Ret-EGFP-R	ctttggcggagcagtctactGTCGAGGGATCTTCATAAGAGAAGAG	#14 & #15: EGFP+polyA amplification for Ret-GFP
#16	cRet left check F1	GGGAAGGGAGAGTTCGATTC	
#17	GFP Rv seq primer 1	AACTTGTGGCCGTTTACGTC	#16 & #17: left-arm knock-in check for Ret-GFP
#18	cRet Right check R1	GGAGGGAAGTTGCTCAACAC	
#19	PolyA Fw seq primer 1	CACTCGGAAGGACATATGGG	#18 & #19: right-arm knock-in check for Ret-GFP
#20	cRet Genome check F1	CCGGTGACAGCCAATGTAAG	
#21	cRet Genome check R1	CCGGTGACAGCCAATGTAAG	#20 & #21: WT allele check for Ret-GFP
#22	pLSODN-4D+Osr1-Left-F	atgcctgcaggctcttcgatTGTGAAAGAGCCCTACCGC	
#23	GFP+Osr1-Left-R	tcctcgcccttgctcaccatTTCTGTCGCTGGGAACCG	#22 & #23: left-arm amplification for Osr1-GFP
#24	Osr1-Left+GFP-F	tgcggttcccagcgacagaaATGGTGAGCAAGGGCGAG	
#25	Osr1-Right+GFP-R	gcagtgctgcagtgtcgcacGTCGAGGGATCTTCATAAGAGAAGAG	#24 & #25: EGFP+polyA amplification for Osr1-GFP
#26	GFP+Osr1-Right-F	tcttatgaagatccctcgacGTGCGACACTGCAGCACTG	
#27	pLSODN-4D+Osr1-Right-R	gtggcaatgcccgggatgatTGAGTTGAGTACCGCCTTTTGG	#26 & #27: right-arm amplification for Osr1-GFP
#28	hCre+Osr1-Left-R	ttcctcttcttcttgggcatTTCTGTCGCTGGGAACCG	#22 & #28: left-arm amplification for Osr1-Cre
#29	polyA+Osr1-Right-F	gatccctcgacctgcagcccGTGCGACACTGCAGCACTG	#27 & #29: right-arm amplification for Osr1-Cre
#30	hCre-F	ATGCCCAAGAAGAAGAGGAAGGTG	
#31	polyA-R	GGGCTGCAGGTCGAGGGATC	#30 & #31: hCre+polyA amplification for Osr1-Cre
#32	Check Osr1 Left F	AATTTCCCATGCATCCTGAG	#17 & #32: left-arm knock-in check for Osr1-GFP
#33	hCre check R1	AGCATCTTCCAGGTGTGCTC	#17 & #33: left-arm knock-in check for Osr1-Cre
#34	Check Osr1 Right R	CGGAGTTTTCGTTGTGTGTG	#19 & #33: right-arm knock-in check for Osr1-GFP and Osr1-Cre
#35	Osr1 genome check R1	GCGAGGCTTGGTCTTAAGTG	#17 & #35 & #36 : genotype for Osr1-GFP
#36	Osr1 genome check F1	TATGTTGAGGGGGCAGTAGG	#33 & #35 & #36 : genotype for Osr1-Cre
#37	rDdx3y 5'-Fw2	accatgattacgccaagctCCTTGACCAGCAAGTGAGTTGG	
#38	rDdx3y 5'-Rev1	tcgacctgcagcccaagctCAGTGGTAGTGCAGGCACAC	#37 & #38: 5'-arm amplification for rDby-RFP
#39	rDdx3y 3'-Fw1	gattactattaataactagtcaataatcaatgtcCTTGACCTTCCCACCTCCCC	
#40	rDdx3y 3'-Rev1	gacattgattattgactagAAGCTCTTCTGATGTCCAAGGGTC	#39 & #40: 3'-arm amplification for rDby-RFP
#41	rDdx3y _sgRNA-F	caccGCACTACCACTGAATCCGCT	
#42	rDdx3y_sgRNA-R	aaacAGCGGATTCAGTGGTAGTGC	

Table S8. Primer information for RT-PCR analysis, related to STAR Methods.

ID	oligo name	sequence
#1	RT-mGapdh F	CATTTGCAGTGGCAAAGTGGAG
#2	RT-mGapdhR	CGTCAGATCCACGACGGAC
#3	RT-mrSix2 F	TGTGGCTCAAGGCGCACTAC
#4	RT-mSix2 R	ACGGGTAGGGGTTGTGAGCG
#5	RT-rSix2 R	CGACGGGTAAGGGTTGTGC
#6	RT-mRet F	AGAGTGAGTTACGAGACCTGCTG
#7	RT-mRet R	CAATCTTGCGGCTGTCACGG
#8	RT-rRet F	GGATCCACATCGATGCGGGC
#9	RT-rRet R	GCACAGCCTGGCCAATGACAC
#10	RT-mFoxd1 F	TCAAGATCCCGCGCGAACCG
#11	RT-mFoxd1 R	CCGCGTGGGGAGCGAGTAG
#12	RT-rFoxd1 F	CGACCGCCGGTGCACACGC
#13	RT-rFoxd1 R	CAGCCGCTTGCACGGCGCAG
#14	RT-mouse Pecam F	TGACTTCCAGACTCTCGAGG
#15	RT-mouse Pecam R	TACTCGACAGGATGGAAATCAC
