# Supplementary material for: Rat cell-derived kidney generation via interspecies blastocyst complementation in an Osr1-KO mouse model
Source: Stem Cell Reports. 2026 Jun 11;21(7):102957. doi: 10.1016/j.stemcr.2026.102957 (PMC13385422; doi:10.1016/j.stemcr.2026.102957)
Supplement: Document S2. Article plus supplemental information [file mmc2.pdf]

STEM CELL REPORTS
Article
OPEN ACCESS

Rat cell-derived kidney generation via interspecies blastocyst complementation in an *Osr1*-KO mouse model

Shunsuke Yuri[1,2,4,*] and Ayako Isotani[2,3,*]
[1]Laboratory of Experimental Animals, Research Institution, National Center for Geriatrics and Gerontology, 7-430 Morioka-cho, Obu, Aichi 474-8511, Japan
[2]Division of Biological Science, Graduate School of Science and Technology, Nara Institute of Science and Technology, 8916-5 Takayama-cho, Ikoma, Nara 630-0192, Japan
[3]Life Science Collaboration Center (LiSCo), Nara Institute of Science and Technology, 8916-5 Takayama-cho, Ikoma, Nara 630-0192, Japan
[4]Lead contact
*Correspondence: shunsukeyuri@ncgg.go.jp (S.Y.), isotani@bs.naist.jp (A.I.)
https://doi.org/10.1016/j.stemcr.2026.102957

SUMMARY

Interspecies blastocyst complementation (BC) provides a promising approach to generate transplantable organs from pluripotent stem cells (PSCs). However, interspecies kidney generation has remained largely unsuccessful, particularly when using rat PSCs in mouse hosts. Here, we investigated multiple renal-deficient models (*Sall1*-, *Ret*-, and *Osr1*-knockouts [KOs]) through reverse BC (rBC) analyses. We identified *Osr1*-KO embryos as lacking both nephron progenitor and ureteric bud lineages, providing a vacant developmental niche for renal reconstruction. Injection of rat embryonic stem cells (ESCs) into *Osr1*-KO mouse blastocysts led to robust rat cell contribution and the formation of kidneys predominantly composed of rat in the vacant niche. These findings establish the *Osr1*-KO model as a permissive platform for interspecies kidney organogenesis and offer mechanistic insight into developmental compatibility underlying xenogeneic organ generation.

INTRODUCTION

The kidneys are essential for maintaining homeostasis by filtering metabolic waste and regulating water, electrolytes, and pH balance (McMahon 2016). Chronic damage from conditions such as diabetes or hypertension leads to irreversible loss of kidney function, as renal tissue has limited regenerative capacity. Kidney transplantation is the most effective treatment for end-stage renal disease, offering superior outcomes to dialysis (Wolfe et al., 1999). However, its use is limited by donor shortages, recipient eligibility, and the complications associated with immunosuppressive therapy (Pascual et al., 2002). While the use of genetically modified porcine organs offers a compelling strategy to mitigate the global organ shortage, substantial immunological challenges associated with human xenotransplantation have yet to be overcome (Griffith et al., 2022; Montgomery et al., 2022; Zhou et al., 2022). The advent of induced pluripotent stem cells (iPSCs) has propelled efforts to generate functional organs from patient-derived cells (Takahashi and Yamanaka et al., 2006; Takahashi et al., 2007). In particular, the generation of transplantable kidneys from pluripotent stem cells (PSCs), including embryonic stem cells (ESCs) and iPSCs, represents an attractive strategy. However, despite substantial progress in deriving renal structures *ex vivo* from PSCs (Freedman et al., 2015; Takasato et al., 2015; Taguchi and Nishinakamura, 2017; Tanigawa et al., 2022), the generation of fully functional, three-dimensional, and size-matched kidneys remains a major challenge.

To solve the problems, the blastocyst complementation (BC) method is a promising technique to generate three-dimensional organs from PSCs (Barlabé et al., 2025; Bigliardi et al., 2025). In this method, PSCs are injected into blastocysts of organ-deficient animals, allowing the PSCs to occupy the vacant developmental niche and form the missing organ. BC has since been applied in intraspecies models to generate not only kidneys but also forebrain, hematoendothelial tissues, lungs, thyroids, and livers (Usui et al., 2012; Matsunari et al., 2020; Chang et al., 2018; Hamanaka et al., 2018; Ran et al., 2020; Ruiz-Estevez et al., 2021; Wen et al., 2021; Miura et al., 2023). Interspecies BC using mouse-rat combinations has also succeeded in producing organs such as pancreas, thymus, kidney, germ cells, lung, heart, forebrain, and sensor neurons (Kobayashi et al., 2010, 2021; Isotani et al., 2011; Yamaguchi et al., 2017; Goto et al., 2019; Zvick et al., 2022; Coppiello et al., 2023; Yuri et al., 2024a, 2024b; Huang et al., 2024; Throesch et al., 2024). Notably, mouse PSCs can generate kidneys in kidney-deficient rat hosts, but there have been no reports on the generation of kidneys derived from rat PSCs in mouse host (Usui et al., 2012; Goto et al., 2019), indicating a directional limitation in interspecies kidney generation.

The mammalian kidney is a highly complex organ composed of numerous distinct cell types (McMahon 2016; Combes et al., 2019). It develops from the metanephros, which arises from the posterior intermediate mesoderm (IM) around embryonic day 10.5 (E10.5) in mice (Saxén 1987; Sukhatme 2003). Kidney organogenesis begins with glial cell line-derived neurotrophic factor

Published by Elsevier Inc. on behalf of International Society for Stem Cell Research.
This is an open access article under the CC BY license (http://creativecommons.org/licenses/by/4.0/).

(GDNF) expression in the metanephric mesenchyme (MM), inducing ureteric bud (UB) outgrowth from the nephric duct (Moore et al., 1996; Pichel et al., 1996). Reciprocal interactions between the UB and the cap mesenchyme (CM)—which contains Six2+ nephron progenitor cells (NPCs)—are essential for nephron induction and NPC self-renewal (Boyle et al., 2008; Kobayashi et al., 2008; Costantini and Kopan 2010). With each UB branching, subsets of CM cells undergo mesenchymal-to-epithelial transition (MET) to form renal vesicles, which develop into nephrons (Carroll et al., 2005). Adjacent to the CM is a population of Foxd1+ stromal progenitors, which generate interstitial lineages—including renin cells, smooth muscle cells, perivascular fibroblasts, and pericytes (Hatini et al., 1996; Sequeira-Lopez et al., 2015)—and support NP maintenance (Boivin et al., 2015).

Sall1 plays a crucial role in kidney organogenesis by maintaining the self-renewal capacity and undifferentiated state of NPCs within the CM (Nishinakamura et al., 2001). *Sall1* is highly expressed in the CM, and its loss results in severe renal hypoplasia or agenesis due to impaired NPC maintenance and defective UB branching. The *Ret* gene encodes the receptor tyrosine kinase RET, which is essential for the initiation and branching morphogenesis of the UB. Upon binding its ligand Gdnf, Gdnf-Ret signaling promotes UB outgrowth from the nephric duct and supports iterative branching within the MM. Disruption of the *Ret* gene leads to failure of UB induction and subsequent kidney agenesis, highlighting its pivotal role in early kidney induction and morphogenesis (Schuchardt et al., 1994, 1996). *Osr1* is a transcription factor expressed broadly in the IM that later becomes restricted to the MM. *Osr1* functions upstream of several key kidney developmental regulators, including *Pax2*, *Six2*, *Eya1*, and *Gdnf* (James et al., 2006). *Osr1* is essential for the specification and survival of NPCs, and its loss leads to apoptosis of the MM and complete absence of kidney formation. Thus, *Osr1* acts as a master regulator of early kidney lineage commitment.

In this study, we aimed to generate rat PSC-derived kidneys using the interspecies BC method in a mouse model, which is not achieved in the previous study (Usui et al., 2012). To establish a suitable organ-deficient host for kidney generation, we evaluated mouse kidney knockout (KO) models such as *Sall1*-KO, *Ret*-KO, and *Osr1*-KO, using the reverse BC (rBC) method—in which genetically modified PSCs are injected into wild-type (WT) blastocysts to assess chimeras composed of mutant and wild-type cells during organogenesis (Yuri et al., 2024a, 2024b)—to determine their suitability and the conditions required for kidney generation. Finally, we successfully generated rat-derived kidneys in the kidney-deficient mouse model via the interspecies BC method.

RESULTS

Analysis of the *Sall1*-deficient mouse model in the rBC method

To elucidate the requisite conditions for kidney generation using the BC method, we first investigated the *Sall1*-KO model previously described in kidney generation with the BC method (Usui et al., 2012; Goto et al., 2019). To investigate the *Sall1*-KO model with the rBC method (Figure 1A), we used mutant mouse ESCs constitutively expressing red fluorescent protein (RFP), similar to our previous report (Yuri et al., 2024a, 2024b). We designed two guide RNAs (gRNAs) to excise the entire exon regions of the *Sall1* gene, thereby establishing *Sall1*-KO mouse ESCs (Figure S1A). The successful excision of the *Sall1* gene was validated via PCR and sequencing analyses (Figure S1B). Seven *Sall1*-KO ESC lines were used to analyze the chimeras (Figure S1C). *Sall1*-KO mouse ESCs were injected into WT mouse embryos (donor: mouse; host: mouse) and analyzed at E14.5 (Table S1). Chimeras comprising 1.4%–16.6% WT (RFP-) cells were not able to develop kidneys (n = 11) (Figures 1B and S1D), mirroring the phenotype observed in *Sall1*-KO mice (Nishinakamura et al., 2001). Conversely, chimeras containing 7.2%–97.2% WT (RFP−) cells exhibited 2 kidneys (n = 41), suggesting that a minimum threshold of 7.2%–16.6% WT cell contribution to the chimeras is required for kidney generation in the *Sall1*-KO model (Figure 1B). In the WT ESC (RFP+) injection analysis, all of the chimeras containing 0.7%–98.6% WT (RFP-) cells contained 2 kidneys (n = 33) (Figure 1B). Immunofluorescence analysis of *Sall1*-KO (RFP+) cells within renal component tissues demonstrated that *Sall1*-KO cells were incapable of contributing to Six2+ NPCs (Figure 1C). Nevertheless, *Sall1*-KO cells were able to contribute into E-Cad+ UB cells in the peripheral region of the kidney, Pbx1+ stromal-derived cells, and Endomucin+ endothelial cells (Figures 1C and 1D). These findings suggest that WT cells predominantly contributed to the nephron progenitor population in the *Sall1*-KO model.

Analysis of *Ret*- and *Ret/Sall1*-deficient models in the rBC method

Next, we focused on the UB tissue, another key component of kidney development. Considering less competitive ability of *Ret*-KO cells relative to WT cells within UB tissue (Shakya et al., 2005; Riccio et al., 2016), we also undertook

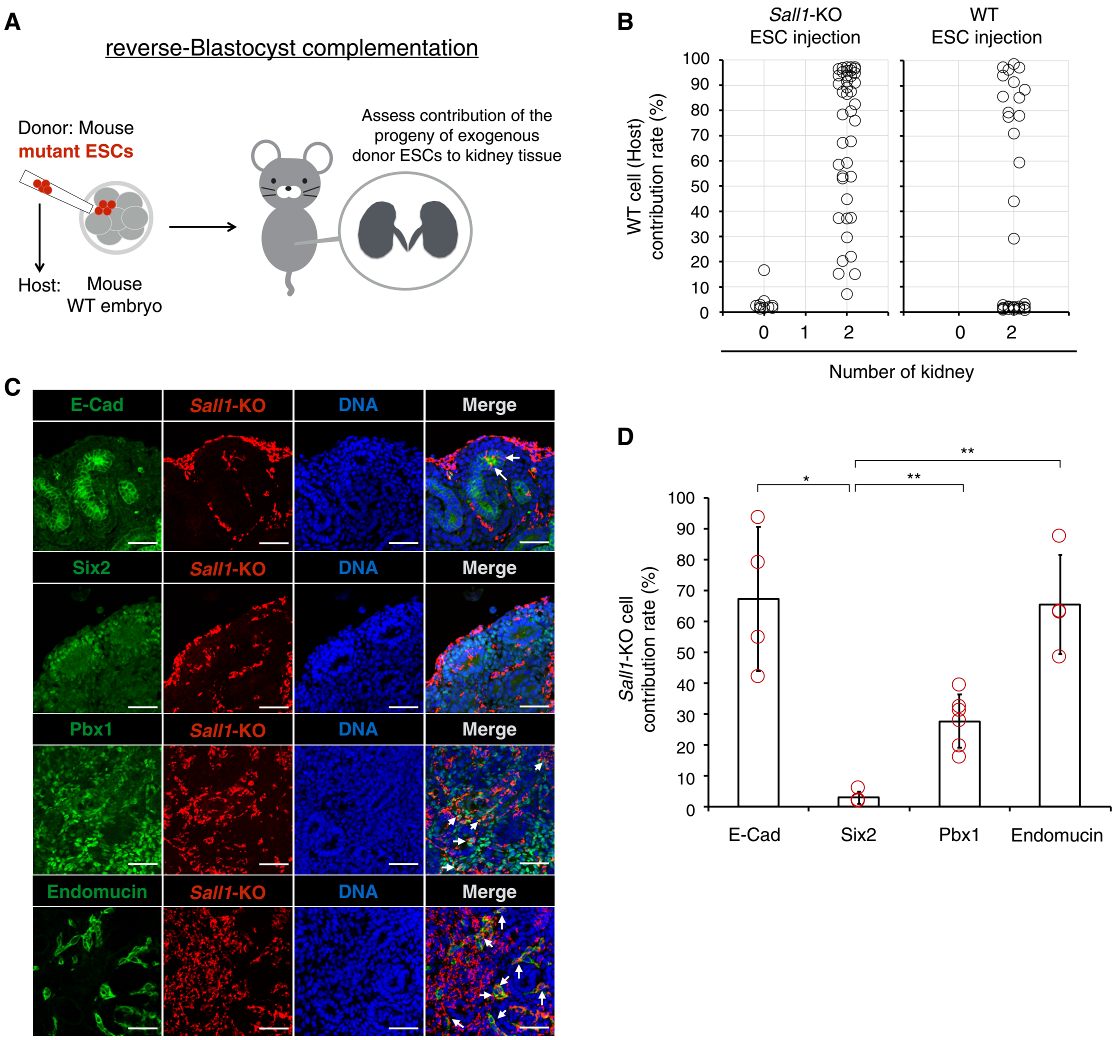

Figure 1. Analysis of the *Sall1*-KO model in kidney with the reverse blastocyst complementation method

(A) Schematic of the reverse-blastocyst complementation (rBC) method. *Sall1*-knockout (KO) mouse embryonic stem cells (ESCs) expressing red fluorescent protein (RFP) were injected into the mouse wild-type (WT) embryo (donor: mouse; host: mouse). Chimeras derived from *Sall1*-KO and WT cells were dissected to determine whether the kidney was present.

(B) Relationship between the cellular contribution rate of the WT cells (host) in tail and the number of the kidney in WT-*Sall1*-KO ESC chimera or WT-WT ESC chimera. WT-*Sall1*-KO ESC chimera without kidney (n = 11) and with kidney (n = 41), or WT-WT ESC chimera with kidney (n = 37) were analyzed.

(C) Representative immunostaining image of E-Cad, Six2, Pbx1, and Endomucin in kidney of WT-*Sall1*-KO ESC chimeras. White arrows indicate that *Sall1*-KO cells localized at E-Cad, Pbx1, and Endomucin-positive cells, respectively. Scale bars, 50 μm.

(D) Quantification of *Sall1*-KO cell labeling in Six2+, E-Cad+, Pbx1+, and Endomucin+ cell populations (4–6 different nonoverlapped regions were analyzed in each marker, n = 2 chimeras). Statistical analyses: unpaired Student's t test, significance at $^{*}p < 0.05$, $^{**}p < 0.01$. Error bars represent mean ± standard deviation (SD).

an analysis using the *Ret*-KO model in the rBC method. To establish *Ret*-KO mouse ESCs, the GFP gene was introduced into the *Ret* gene locus (Figure S2A), resulting in the establishment of three distinct *Ret*-KO ESC clones (Figures S2B–S2D). When *Ret*-KO ESCs were injected into WT mouse embryos (donor: mouse; host: mouse) and analyzed at E14.5 (Table S2), chimeras comprising 0.8%–2.6% WT cells were unable to develop kidneys (n = 13), reflecting the phenotype observed in *Ret*-KO mice (Schuchardt et al., 1994, 1996) (Figure 2A). On the other hand, chimeras containing 2.1%–12.9% WT cells exhibited 1 kidney (n = 4), and chimeras containing 16.3%–98.9% WT cells exhibited 2 kidneys (n = 49) (Figure 2A). These results indicate that a minimum threshold of approximately

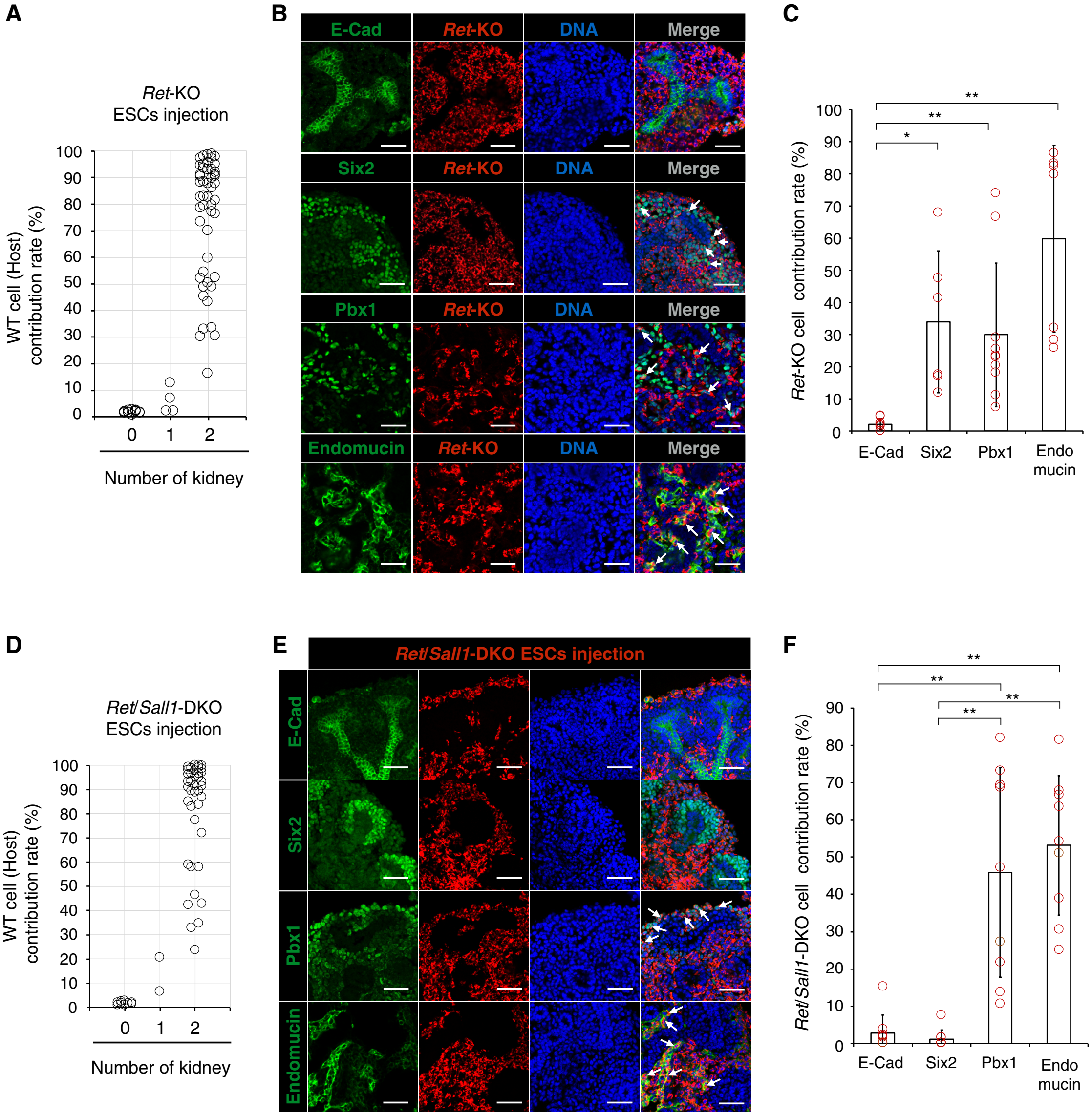

Figure 2. Analysis of *Ret*-KO and *Ret/Sall1*-DKO models in kidney using the rBC method

(A) Relationship between the cellular contribution rate of the WT cells (host) in tail and the number of the kidney in WT-*Ret*-KO ESC chimeras. WT-*Ret*-KO ESC chimeras without kidney (n = 13), with 1 kidney (n = 4), and with 2 kidneys (n = 53) were analyzed.

(B) Representative immunostaining image of E-Cad, Six2, Pbx1, and Endomucin in kidney of WT-*Ret*-KO ESC chimeras. White arrows indicate that *Ret*-KO (RFP+) cells localized at Six2, Pbx1, or Endomucin-positive cells, respectively. Scale bars, 50 μm.

(C) Quantification of *Ret*-KO cells labeling in Six2+, E-Cad+, Pbx1+, and Endomucin+ cell population (6–10 different nonoverlapped regions were analyzed in each marker, n = 2 chimeras). Statistical analyses: unpaired Student's t test, significance at $*p < 0.05$, $**p < 0.01$. Error bars represent mean ± standard deviation (SD).

(D) Relationship between the cellular contribution rate of the WT cells (host) in tail and the number of the kidney in WT-*Ret/Sall1*-DKO ESC chimeras. WT-*Ret/Sall1*-DKO ESC chimeras without kidney (n = 9), with 1 kidney (n = 2), and with 2 kidneys (n = 38) were analyzed.

(E) Representative immunostaining image of E-Cad, Six2, Pbx1, and Endomucin in kidney of *Ret/Sall1*-DKO and WT cell chimeras. White arrows indicate that *Ret/Sall1*-KO cells localized at Pbx1 or Endomucin-positive cells, respectively. Scale bars, 50 μm.

(F) Quantification of *Ret/Sall1*-DKO cell labeling in Six2+, E-Cad+, Pbx1+, and Endomucin+ cell populations (9 different nonoverlapped regions were analyzed in each marker, n = 3 chimeras). Statistical analyses: unpaired Student's t test, significance at $**p < 0.01$. Error bars represent mean ± standard deviation (SD).

2% of WT cells is required for at least one of the kidneys to develop in the *Ret*-KO model. Immunofluorescence analysis showed that *Ret*-KO cells were not specifically observed in E-Cad+ UB tissues (Figures 2B and 2C). Conversely, *Ret*-KO cells were capable of contributing to NPCs, as well as to stromal and endothelial tissues (Figures 2B and 2C). Together, these results demonstrate that WT cells predominantly complement UB-derived tissues in the kidneys of the *Ret*-KO model.

To further assess the efficacy of the rBC method in a two-gene KO model, we generated *Ret*/*Sall1* double-KO (DKO) mouse ESCs. We introduced mutations into the *Ret* gene within *Sall1*-KO ESCs and successfully established three *Sall1*/*Ret*-DKO ESC lines (Figure S2E). When these *Ret*/*Sall1*-DKO ESCs were injected into WT mouse embryos (donor: mouse; host: mouse) and analyzed at E14.5 (Table S2), it was determined that chimeras containing 0.9%–2.7% WT cells exhibit 0 kidney (n = 9), while the chimeras containing 6.4% or 20.6% showed 1 kidney (n = 2) and chimeras containing 23.7%–99.9% of WT cells exhibited 2 kidneys (n = 38) (Figure 2D). Notably, *Ret*/*Sall1*-DKO cells failed to contribute to either NPCs (Six2+) or UB cells (E-Cad+) (Figures 2E and 2F), demonstrating a combined negative effect of the *Sall1*-KO and *Ret*-KO phenotypes. Collectively, these results indicate that the rBC method can effectively elucidate kidney-defective models carrying multiple gene mutations.

Analysis of the *Osr1*-KO model in the rBC method

Given that the *Osr1* gene is expressed in IM cells, which are thought to be progenitors of most of kidney component cells (Mugford et al., 2008), and that the *Osr1*-KO mouse exhibits abnormalities in the nephric duct and NPCs (James et al., 2006), we utilized the rBC method to assess the competitive ability of *Osr1*-KO cells against WT cells during kidney development. Using blastocysts obtained from the *Osr1*-GFP and *Osr1*-Cre mouse lines established in this study (Figures S3A and S3B), we established seven RFP-expressing *Osr1*-KO ESC lines and one WT ESC line. When these *Osr1*-KO mouse ESCs were injected into WT mouse embryos (donor: mouse; host: mouse) and analyzed at E12.5 (Table S3), two kidneys were observed in the chimeras containing 28.6%–91.7% WT cells (n = 37), one kidney was seen in the chimeras containing 10.6%–32% WT cells (n = 5), and no kidneys were detected when 0.5%–31.3% WT cells (n = 25) were present in the chimera consisting of *Osr1*-KO and WT cells (Figure 3A). In contrast, chimeras containing WT ESC-derived cells consistently showed two kidneys (n = 12) (Figure 3A). Immunofluorescence analysis indicated that *Osr1*-KO cells were conspicuously absent from the UB cells (E-Cad+) and NPCs (Six2+) but were present in the stromal cells (Pbx1+) and endothelial cells (Endomucin+) (Figures 3B and 3C). These results suggest that approximately 10% of WT cells are sufficient to generate at least one kidney and that WT cells predominantly form the NPCs and UB tissues in the kidneys of the *Osr1*-KO model.

Analysis of the *Osr1*-KO model in the BC method

Subsequently, we assessed the *Osr1*-KO model using the intraspecies BC method. RFP-expressing WT mouse ESCs were injected into the *Osr1*-KO blastocysts obtained from *Osr1*-heterozygous mouse crossing (donor: mouse; host: mouse). Due to the inclusion of WT ESC-derived cells in the chimeras, which complicates genotype analysis, two distinct *Osr1* heterozygous mouse lines, *Osr1*-GFP and *Osr1*-Cre, were used for the intraspecies BC method (Figure 4A). When both *Osr1*-GFP and *Osr1*-Cre bands were detected in PCR analysis, the chimera was classified as the *Osr1*-KO genotype (Figures 4B and S3C). When analyzing the chimeras at E12.5–E14.5 (Table S4), 10.8%–85.8% WT cell contribution to the chimeras was necessary to generate two kidneys (n = 17), while 0%–31% WT cell contribution from ESCs exhibited no kidney (n = 11) in the *Osr1*-KO genotype (Figure 4C), suggesting that chimeras containing more than 10% WT cells could sometimes generate kidney, but more than 30% WT cells mostly could generate kidney in the *Osr1*-KO model. In contrast, 2 kidneys were present in the WT (n = 45), *Osr1*-Cre (n = 45), and *Osr1*-GFP (n = 40) genotypes in all embryos with or without WT cell-derived ESCs (Figure 4C). In *Osr1*-GFP embryos, GFP signals were observed in tissues expressing *Osr1* (Figure S3D); however, GFP signals were absent in the kidneys of chimeras with the *Osr1*-KO (Cre/GFP) genotype (Figure 4D), indicating that NPCs expressing *Osr1* were predominantly replaced by WT cells derived from the injected ESCs. To further examine kidney tissues, we performed immunostaining for E-Cad, Six2, Pbx1, and Endomucin in kidneys from the *Osr1*-KO genotype chimeras. Immunostaining analysis revealed that RFP-expressing WT cells were predominant in nearly all kidney tissues; however, detecting host cells that lacked fluorescent protein expression proved challenging (Figure 4E). To determine which tissues were complemented by the injected WT ESCs in the kidney, we sorted the RFP-positive population derived from injected ESCs and the RFP-negative population from host cells, conducting reverse-transcription PCR (RT-PCR) analysis (Figure 4F). The analysis showed that expression levels of *Six2* and *Ret* were significantly lower in the RFP-negative cell population in the *Osr1*-KO genotype compared to that in the non-*Osr1*-KO genotype (Figure 4G). Conversely, *Foxd1* and *Pecam1*, endothelial cell markers, did not exhibit significant differences between *Osr1*-KO and non-*Osr1*-KO genotypes (Figure 4G). These findings suggest that the injected WT ESCs effectively reconstituted the

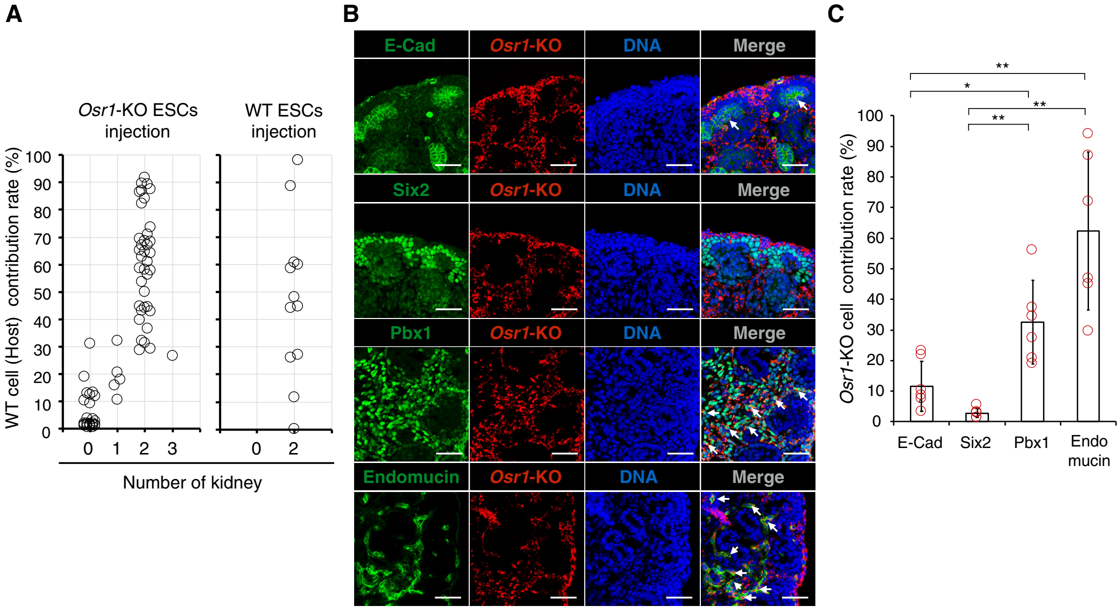

Figure 3. Analysis of the *Osr1*-KO model using the rBC method

(A) Relationship between the cellular contribution rate of the WT cells (host) in tail and the number of the kidney in WT-*Osr1*-KO ESC chimeras or WT-WT ESC chimeras. WT-*Osr1*-KO ESC chimera without kidney ($n = 25$), with 1 kidney ($n = 5$), with 2 kidneys ($n = 37$), and with 3 kidneys ($n = 1$) and WT-WT ESC chimeras with 2 kidneys ($n = 12$) were analyzed.

(B) Representative immunostaining image of E-Cad, Six2, Pbx1, and Endomucin in kidney of *Osr1*-KO and WT cell chimeras. White arrows indicate that *Osr1*-KO cells localized at E-Cad, Pbx1, or Endomucin-positive cells, respectively. Scale bars, 50 μm.

(C) Quantification of *Osr1*-KO cell labeling in Six2+, E-Cad+, Pbx1+, and Endomucin+ cell population (6–7 different nonoverlapped regions were analyzed in each marker, $n = 4$ chimeras). Statistical analyses: unpaired Student's t test, significance at $*p < 0.05$, $**p < 0.01$. Error bars represent mean ± standard deviation (SD).

nephron progenitor and UB compartments in the *Osr1*-KO model, corroborating the outcomes obtained with the rBC method.

Analysis of the *Osr1*-KO model in the interspecies BC method

Given that chimeras comprising over 10% WT cells in the *Osr1*-KO model have demonstrated the capacity to generate at least one kidney, and considering that the rat contribution to the kidney occasionally surpassed this threshold (Figure S4A), we then applied the *Osr1*-KO model to an interspecies BC method to ascertain whether rat ESCs could facilitate kidney formation in the *Osr1*-KO mouse model (Table S5). RFP-positive rat ESCs (rDby-RFP) were injected into blastocysts obtained from *Osr1*-heterozygous mouse crossings (donor: rat; host: mouse), and the resulting chimeras were analyzed at E13.5 (Figures 5A and 5B). Rat chimeras derived from non-*Osr1*-KO genotypes occasionally exhibited fewer than two kidneys, irrespective of the contribution of rat cells in the chimeras (Figures S4B and S4C). We further investigated similar phenomena in chimeras generated by injecting the same rat ESCs into rat embryos (Table S6); however, all rat-rat chimeras consistently developed two kidneys, indicating that the rat ESCs used in this study did not cause any phenotypic alterations in kidney development (Figure S4D). In the chimeras with the *Osr1*-KO genotype, the rat chimeras could develop kidneys only under conditions of higher rat cell chimerism (Figure 5C). When rat ESCs were injected into mouse embryos, the chimerism observed in the kidney mirrored that in the tail, diverging from the pattern seen in the lung (Figure S4A). On the other hand, the distribution of chimerism in the kidney, tail, and lung was relatively uniform when rat ESCs were injected into rat embryos (Figure S4E). Therefore, we deduced that rat cell contribution in the kidney could be predicted from that in the tail, indicating that kidney formation in the *Osr1*-KO model requires a rat cell contribution rate in the tail exceeding 30%–40% (Figure 5D). Similar results, where the chimeras of non-*Osr1*-KO genotypes exhibiting an unexpected number of kidneys and chimeras with the *Osr1*-KO genotype manifesting kidney development only under higher rat chimerism, were observed in the different rat ESCs (rRFP5-2) (Figures S5A–S5D; Table S5). These findings imply that even rat ESCs could complement the kidney-deficient phenotype in mice if the necessary conditions

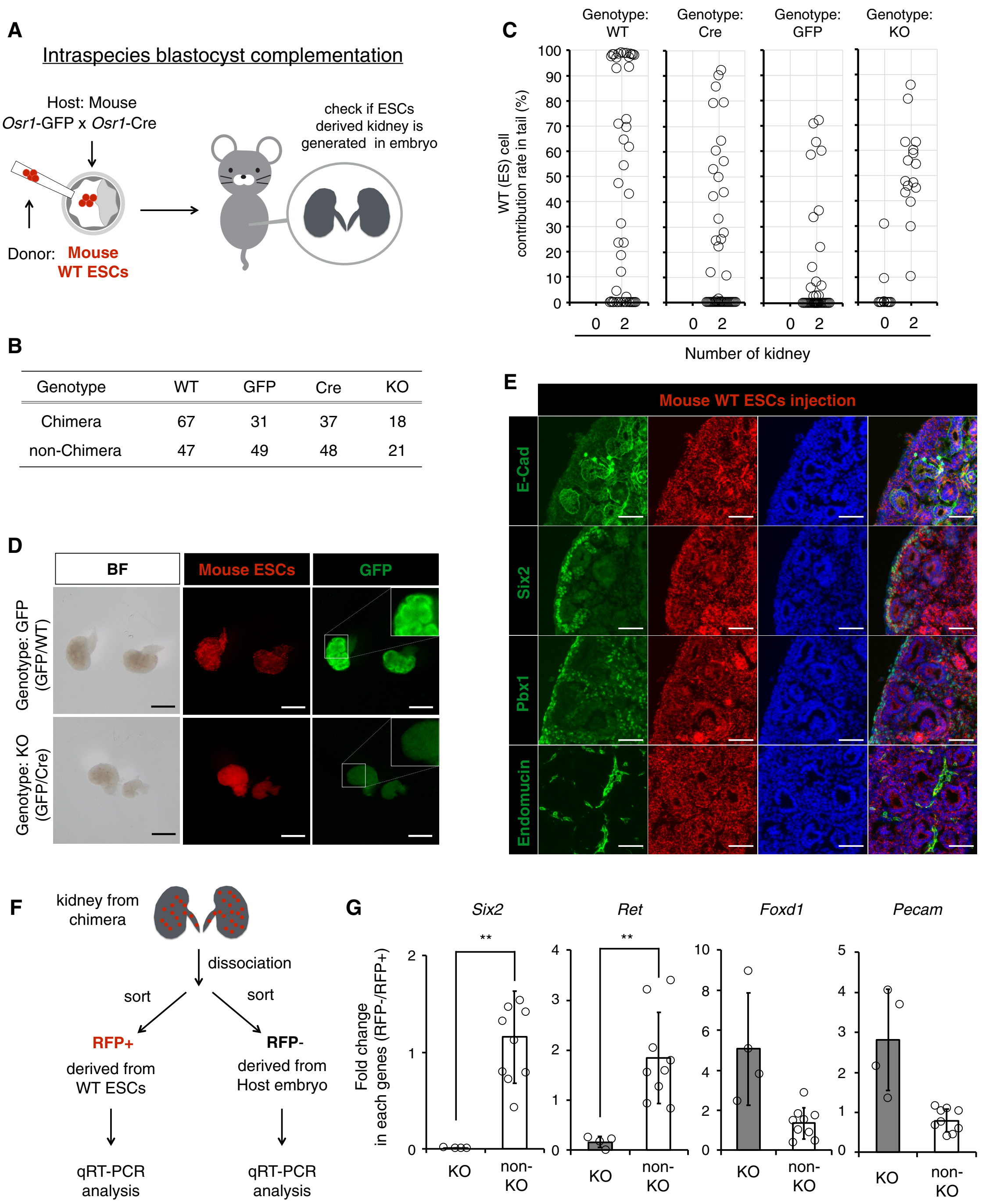

Figure 4. Intraspecies BC method-generated kidney via the *Osr1*-KO mouse model

(A) Schematic of the intraspecies blastocyst complementation (BC) method. Red fluorescent protein (RFP)-expressing mouse WT ESCs were injected into the embryos obtained by crossing *Osr1*-GFP heterozygous mouse and *Osr1*-Cre heterozygous mouse (donor: mouse; host: mouse). Chimeras were dissected at E12.5–E14.5.

(B) Genotype results of intraspecies blastocyst complementation.

(C) Relationship between the cellular contribution rate of the injected WT cells in tail and the number of the kidney in WT-WT ESC chimeras, *Osr1*-Cre-WT ESC chimeras, *Osr1*-GFP-WT ESC chimeras, or *Osr1*-KO-WT ESC chimeras. WT-WT ESC chimeras with 2 kidneys (n = 45), *Osr1*-Cre-WT ESC chimeras with 2 kidneys (n = 45), *Osr1*-GFP-WT ESC chimera with 2 kidney (n = 40), or *Osr1*-KO-WT ESC chimeras with 2 kidneys (n = 17) and 0 kidney (n = 11) were analyzed.

(legend continued on next page)

are fulfilled. To determine whether the reconstructed rat kidneys consisted of NPCs and UB-derived tissues, as observed in the intraspecies BC method, we conducted an immunofluorescence experiment. The reconstituted kidney in the *Osr1*-KO model displayed RFP signals throughout, in contrast to kidneys from the non-*Osr1*-KO model (Figure 5E). Six2+ NPCs and E-Cad+ UB tissues were likely composed of RFP+ rat cells in the kidney obtained from the *Osr1*-KO model compared to that from the non-*Osr1*-KO model (Figure 5E). However, the lack of fluorescence signals in host-derived cells made it challenging to identify where non-fluorescent (host) cells contributed. Further analysis of the rat-derived kidneys from the *Osr1*-KO model using RT-PCR with mouse-specific primers—including NPCs marker *Six2*, UB marker *Ret*, and stroma marker *Foxd1*—revealed minimal detection of *Six2* and *Ret* within the mouse cell population of *Osr1*-KO chimeras. Conversely, *Foxd1* was detected in the mouse cell population of the *Osr1*-KO chimeras, similar to those in the non-*Osr1*-KO chimeras (Figure 5F). Moreover, when examining the proportional contribution of mouse and rat cells across kidney tissue components, we found that NPCs and UB cells were overwhelmingly dominated by rat cells in the *Osr1*-KO chimeras (Figure 5G). Furthermore, the reconstructed rat kidneys exhibited expression of markers of nephron tubules and early nephron development, including *Lotus tetragonolobus* lectin (LTL), WT1, E-cadherin, and Pax2, as well as the endothelial marker CD31, following *in vitro* kidney organ culture to promote maturation (Figures S6A and S6B). These results demonstrate that rat cells can indeed occupy the developmental niche present in *Osr1*-KO mouse models, provided that the conditions are conducive to kidney development.

DISCUSSION

In this study, we have elucidated the requisite conditions for successful kidney generation in various kidney-deficient models using the rBC method. Our results indicate that *Sall1*-KO cells are specifically incapable of contributing to NPCs, *Ret*-KO cells cannot contribute to UB cells, and *Osr1*-KO cells are unable to contribute to both NPCs and UB cells. Consequently, we infer that WT cells can complement these tissues in each respective model when performing the BC method. The required conditions for kidney generation in the *Sall1*-KO, *Ret*-KO, and *Osr1*-KO models were found to vary depending on the model. Notably, the *Ret*-KO model required the fewest WT cells for kidney development compared to the *Sall1*-KO and *Osr1*-KO models. This may be attributed to the occasional presence of hypoplastic kidneys even in *Ret*-KO mice (Schuchardt et al., 1994, 1996). The minimum threshold necessary in kidney generation with the rBC method may reflect the severity of each kidney-deficient model.

The rBC method allowed us to analyze chimeras containing even double-gene KO (*Ret*/*Sall1*-KO) cells with 100% probability, a significant improvement in efficiency compared to the conventional method of crossing heterozygous mice, which yields only 1 in 16 (6.25%) embryos when targeting two genes. Noteworthily, the rBC method has the potential to analyze models with KOs of not only two genes but also multiple genes or complex gene modification for organ deficiencies, as it only requires the establishment of genetically modified ESCs. The implementation of this system would efficiently address the challenge of generating all cells of a target organ with PSC-derived cells.

In previous studies, the kidney was found to be completely absent in *Osr1*-KO mice, although the nephric duct was formed but mildly affected (James et al., 2006). *Osr1* is expressed in IM cells, which are nephric duct precursors, and in NPCs but not in the nephric duct and UB themselves (James et al., 2006). Thus, the nephric duct defects observed in *Osr1*-KO mice have been proposed to arise either from abnormalities in nephric duct precursors or from impaired interactions between the nephric duct and *Osr1*-expressing mesenchyme (Obara-Ishihara et al., 1999). In our analysis of chimeras containing *Osr1*-KO and WT cells, we found that *Osr1*-KO cells could not contribute to UB tissues, which differentiate from the nephric duct. This indicates that *Osr1*-KO cells indeed possess a defect in nephric duct precursor cells, rather than in the interaction between the duct and *Osr1*-expressing mesenchyme. Consequently, *Osr1*-KO cells were

(D) Representative kidney images obtained from *Osr1*-GFP-WT or *Osr1*-KO-WT chimeras. Scale bars, 500 μm.
(E) Representative immunostaining image of E-Cad, Six2, Pbx1, and Endomucin in kidney of *Osr1*-KO and WT cell chimeras. Scale bars, 100 μm.
(F) Schematic of the fluorescence-activated cell sorting experiment. Kidneys obtained from chimeras were dissociated and sorted into RFP+ and RFP− population. RFP+ population derived from injected ESCs and RFP− population derived from host cells. Each sorted RFP+ and RFP− cells were performed with quantitative reverse-transcription polymerase chain reaction (RT-qPCR) analysis.
(G) RT-qPCR analysis of kidney derived from *Osr1*-KO chimeras or non-*Osr1*-KO chimeras to examine the expression of *Six2*, *Ret*, *Foxd1*, and *Pecam*. All data were normalized by *Gapdh*, and each gene expression was compared between the RFP− population and RFP+ populations. Each plot data were obtained from different chimeras (*Osr1*-KO: $n = 4$ chimeras, non-*Osr1*-KO: $n = 9$ chimeras). Error bars represent mean ± standard deviation (SD). $^{**}p < 0.01$; unpaired two-tailed Student's t test.

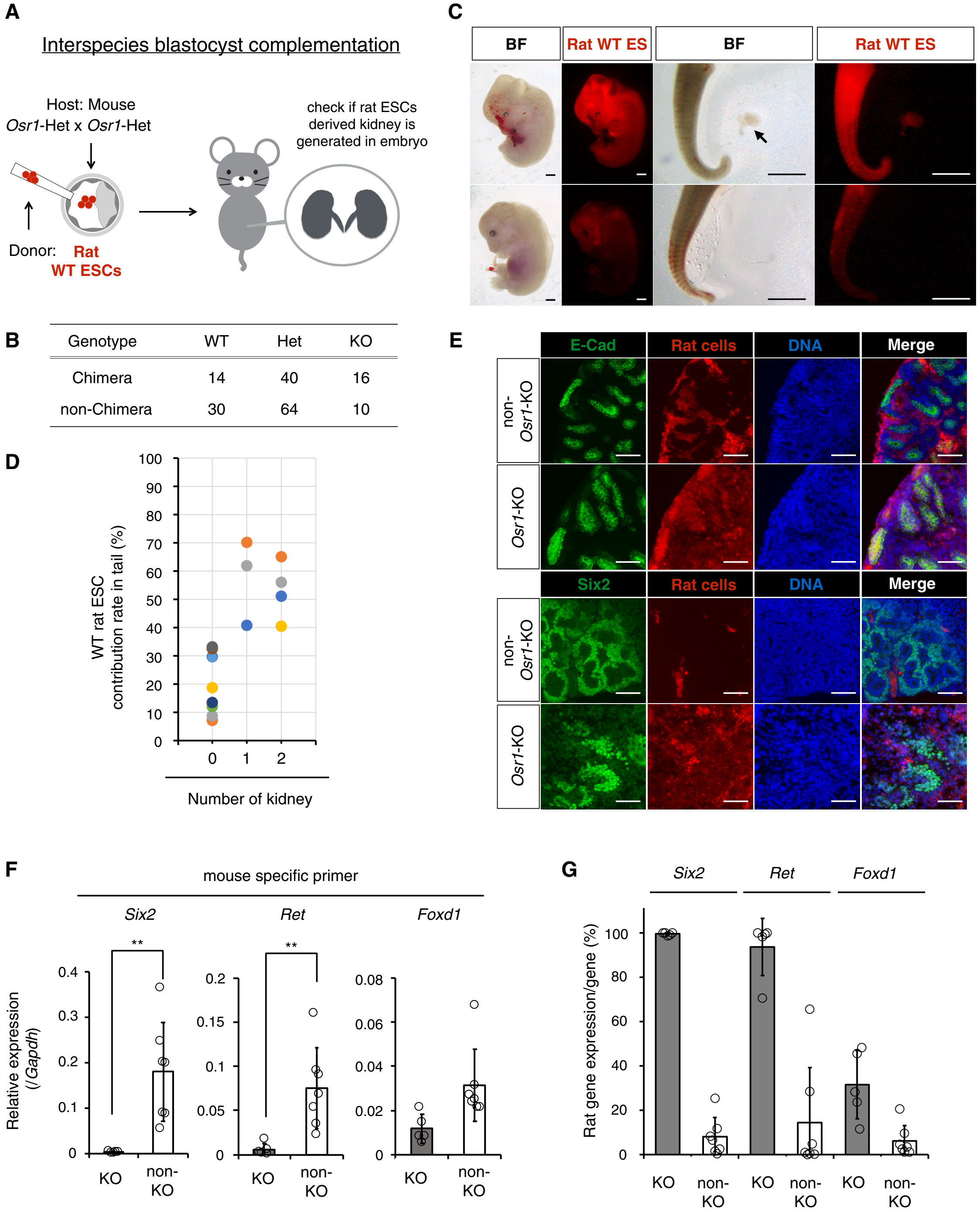

Figure 5. Interspecies BC method for generating rat kidney with the *Osr1*-KO mouse model

(A) Schematic of the interspecies blastocyst complementation (BC) method. RFP-expressing rat ESCs were injected into the embryos obtained by crossing *Osr1* heterozygous male with female mice (donor: rat; host: mouse). Chimeras derived from *Osr1*-KO mouse or non-*Osr1*-KO mouse and wild-type (WT) rat cells were dissected at E13.5.

(B) Genotype results of interspecies blastocyst complementation.

(C) Representative images of embryos and kidneys derived from chimeras generated from *Osr1*-KO embryos and rat ESCs (RFP+: rDby-RFP), showing high rat contribution (upper) and low rat contribution (lower). Chimeras with high rat contribution contained kidneys (black arrow) in the *Osr1*-KO background, and RFP-expressing kidneys were observed. Scale bars: 1 mm.

(legend continued on next page)

outcompeted by WT cells during differentiation into the nephric duct from *Osr1*-positive IM cells.

Interestingly, an abnormal kidney phenotype was rarely observed in mouse-rat interspecies chimeras, where rat ESCs were injected into mouse embryos, similar to the previous study (Yamaguchi et al., 2018). The frequency of these phenotypes did not correlate with the level of rat ESC-derived cell chimerism. Since the phenotype was absent when rat ESCs were injected into rat embryos, it is likely that the abnormalities were not due to intrinsic defects in the injected rat ESCs but rather to incompatibilities between mouse and rat cells. These species-specific incompatibilities may include interspecies cellular interactions, mismatched ligand-receptor interactions, and other intrinsic or extrinsic cellular factors. Thus, the abnormalities in kidney development observed in these mouse-rat chimeras may provide important insights into organogenesis in future interspecies chimeras. A comprehensive understanding of these mechanisms may, for example, allow us to explore unusual interactions between heterologous cells and important pathways for organogenesis.

We successfully achieved rat kidney reconstruction in a mouse kidney-deficient model with the interspecies BC method, contradicting previous reports that deemed this impossible (Usui et al., 2012). The inability of rat cells to develop in the mouse kidney-deficient model was previously attributed to their failure to integrate into MM tissues (Usui et al., 2012). Consistent with these prior observations, our study also found that rat cells rarely contributed to kidney tissues, including Six2+ NPCs. However, we found that rat cells occasionally surpassed the minimum threshold required for kidney generation in the *Osr1*-KO kidney-deficient model. Consequently, we were able to generate rat kidneys in the mouse kidney-deficient model when the contribution of rat cells was sufficiently high. If the difficulty of rat cells to contribute to mouse kidneys is improved, rat kidneys will be more easily created in the future. Since human cells are almost always difficult to contribute to other species organs (Wu et al., 2017; Strell et al., 2022; Wang et al., 2023), analysis and improvement of this problem in mouse-rat chimeras will make it possible to create human organs in xenogeneic animals in the future.

We analyzed the rat-reconstructed kidneys only at E13.5 because chimeras harboring rat kidneys emerged only when rat cell contribution was high in the *Osr1*-KO model, and these high-rat-contribution chimeras did not survive beyond E14.5, likely due to cardiac abnormalities (Yuri et al., 2024a). The previous study (Usui et al., 2012), which discussed that rat cells cannot generate kidneys in the mouse kidney-deficient model, did not specify the timing of their analysis. Therefore, it is possible that chimeras containing rat kidneys may not have survived if analyzed later than E14.5. Another potential explanation for the absence of rat kidneys in the mouse kidney-deficient model in previous studies is the use of the *Sall1*-KO mouse model, which is deficient only in MM tissues. While rat cells can complement MM tissues in the *Sall1*-KO model, rat cells cannot completely complement to UB tissues, possibly leading to unsuitable interspecies interactions. Conversely, the *Osr1*-KO model used in this study is predominantly deficient in both MM and UB lineages, allowing rat cells to complement both lineages and thereby facilitating proper tissue interactions. Future studies employing ablation strategies targeting both MM and UB tissues will be important to extend and generalize this hypothesis beyond the *Osr1*-KO model used in this study.

In this study, the analyses were restricted to embryonic stages (up to E13.5), as embryos with high levels of rat cell chimerism exhibited embryonic lethality, precluding the evaluation of postnatal maturation and renal function. The requirement for a high proportion of rat cell contribution in the kidney indicates that developmental incompatibility and interspecies cell competition continue to impede efficient complementation. Furthermore, the long-term structural and functional fidelity of the reconstructed kidneys, including nephron segmentation and urinary tract connectivity, has yet to be assessed. Future studies will be required to determine whether BC-derived kidneys can undergo vascularization, structural maturation, and functional integration following transplantation into host animals, as demonstrated for embryonic kidney rudiments at comparable developmental stages (Dekel et al., 1997, 2002, 2003). Finally, although the *Osr1*-KO model predominantly lacks both MM and UB lineages, it

(D) Relationship between the cellular contribution rate of the injected rat WT cells in tail and the number of the kidney in Osr1-KO and WT rat ESC chimeras. *Osr1*-KO-rat WT ESC chimeras with 2 kidneys (n = 4), 1 kidney (n = 3), and 0 kidney (n = 9) were analyzed.

(E) Representative immunostaining image of E-Cad and Six2 in kidney of *Osr1*-KO and rat WT cell chimera or non-*Osr1*-KO and rat WT cell chimera. The kidney tissues were organ cultured for 5 days. Scale bars, 100 μm.

(F) Quantitative reverse transcription polymerase chain reaction (RT-qPCR) analysis results for mouse *Six2*, *Ret*, and *Foxd1* in the kidney derived from rDby-RFP chimeras and rRFP5-2 chimeras. Data were normalized to mouse *Gapdh* expression levels. Samples were extracted from the kidneys of chimeras. All values are expressed as mean ± standard deviation from at least triplicate experiments (*Osr1*-KO: n = 5 kidneys, non-KO: n = 7 kidneys). $^{**}p < 0.01$; unpaired two-tailed Student's t test.

(G) Variation in the presence of rat cells in each kidney tissue component. Note that rat *Six2* and *Ret* were predominantly detected compared with mouse genes but not rat *Foxd1* in the rat *Osr1*-KO chimera (*Osr1*-KO: n = 5 kidneys, non-KO: n = 7 kidneys).

may not represent a completely anephric state, as non-MM/UB components such as stromal or endothelial cells could persist and influence the patterning of complemented tissues. Alternative or conditional kidney-deficient models may be necessary to achieve a fully anephric state and refine the developmental niche for renal complementation. To assess potential host cell contribution, single-cell and spatially resolved analyses will also be important to more precisely define donor-host contributions across renal compartments. Addressing these limitations will be critical for evaluating the functional potential of interspecies kidney generation and improving cross-species developmental compatibility.

RESOURCE AVAILABILITY

Lead contact

Further information and requests for resources and reagents should be directed to and will be fulfilled by the lead contact, Shunsuke Yuri (shunsukeyuri@ncgg.go.jp).

Materials availability

Cell lines and mouse lines generated in this study are available with a material transfer agreement. Requests should be directed to the lead contact.

Data and code availability

- This study did not generate any unique dataset.
- This study did not generate any original code.
- All used software is listed in the key resources table. Any additional information required to reanalyze the data reported in this paper is available from the lead contact upon reasonable request.

ACKNOWLEDGMENTS

We thank the members of Isotani Laboratory in NAIST and the members of Laboratory of Experimental Animals in NCGG for their helpful assistance and discussions. The LiSCO at the Nara Institute of Science and Technology (NAIST) and the core facility at National Center for Geriatrics and Gerontology (NCGG) were instrumental in this study. We appreciate Dr. Masahito Ikawa (Osaka University) for kindly providing the pCX-EGFP plasmid, pCX-hCre plasmid, R01 ESC, and rGBGS #6 (F344) rats. This study was supported by grants from the Japan Society for the Promotion of Science KAKENHI (grant nos. 23K18577 and 24K01948 to A.I. and 18K06031, 22K06067, and 25K02202 to S.Y.), KAC 40th Anniversary Research grant to A.I., the Novartis Foundation (Japan) for the Promotion of Science to A.I., and the Foundation for Nara Institute of Science and Technology to S.Y., and the Research Fund for Longevity Sciences from the NCGG (24–31).

AUTHOR CONTRIBUTIONS

Conceptualization, S.Y. and A.I.; methodology, S.Y.; validation, S.Y. and A.I.; formal analysis, S.Y.; investigation, S.Y. and A.I.; writing – original draft, S.Y.; writing – review and editing, S.Y. and A.I.; visualization, S.Y.; supervision, A.I.; funding acquisition, S.Y. and A.I.

DECLARATION OF INTERESTS

The authors declare no competing interests.

STAR★METHODS

Detailed methods are provided in the online version of this paper and include the following:

- KEY RESOURCES TABLE
- EXPERIMENTAL MODEL AND STUDY PARTICIPANT DETAILS
  - Animals
  - Cell culture
- METHOD DETAILS
  - Plasmid construction
  - Genotyping
  - ESCs injection
  - Flow cytometry analysis and fluorescence-assisted cell sorting
  - RNA expression analysis
  - Immunocytochemistry staining
- QUANTIFICATION AND STATISTICAL ANALYSIS
  - Statistical analysis

SUPPLEMENTAL INFORMATION

Supplemental information can be found online at https://doi.org/10.1016/j.stemcr.2026.102957.

Received: December 15, 2025
Revised: May 12, 2026
Accepted: May 13, 2026
Published: June 11, 2026

REFERENCES

Barlabé, P., Aranguren, X.L., and Coppiello, G. (2025). Blastocyst complementation: current progress and future directions in xenogeneic organogenesis. Stem Cell Res. Ther. *16*, 321. https://doi.org/10.1186/s13287-025-04426-y.

Bigliardi, E., Shetty, A.V., Low, W.C., and Steer, C.J. (2025). Interspecies blastocyst complementation and the genesis of chimeric solid human organs. Genes *16*, 215. https://doi.org/10.3390/genes16020215.

Boivin, F.J., Sarin, S., Lim, J., Javidan, A., Svajger, B., Khalili, H., and Bridgewater, D. (2015). Stromally expressed β-catenin modulates Wnt9b signaling in the ureteric epithelium. PLoS One *10*, e0120347. https://doi.org/10.1371/journal.pone.0120347.

Boyle, S., Misfeldt, A., Chandler, K.J., Deal, K.K., Southard-Smith, E.M., Mortlock, D.P., Baldwin, H.S., and de Caestecker, M. (2008). Fate mapping using Cited1-CreERT2 mice demonstrates that the cap mesenchyme contains self-renewing progenitor cells and gives rise exclusively to nephronic epithelia. Dev. Biol. *313*, 234–245. https://doi.org/10.1016/j.ydbio.2007.10.014.

Carroll, T.J., Park, J.S., Hayashi, S., Majumdar, A., and McMahon, A.P. (2005). Wnt9b plays a central role in mesenchymal-to-epithelial transitions underlying organogenesis. Dev. Cell *9*, 283–292. https://doi.org/10.1016/j.devcel.2005.05.016.

Chang, A.N., Liang, Z., Dai, H.Q., Chapdelaine-Williams, A.M., Andrews, N., Bronson, R.T., Schwer, B., and Alt, F.W. (2018). Neural blastocyst complementation enables mouse forebrain organogenesis. Nature *563*, 126–130. https://doi.org/10.1038/s41586-018-0586-0.

Choi, J., Huebner, A.J., Clement, K., Walsh, R.M., Savol, A., Lin, K., Gu, H., Di Stefano, B., Brumbaugh, J., Kim, S.Y., et al. (2017). Prolonged Mek1/2 suppression impairs the developmental potential of embryonic stem cells. Nature *548*, 219–223. https://doi.org/10.1038/nature23274.

Combes, A.N., Phipson, B., Lawlor, K.T., Dorison, A., Patrick, R., Zappia, L., Harvey, R.P., Oshlack, A., and Little, M.H. (2019). Single cell analysis of the developing mouse kidney provides deeper insight into marker gene expression and ligand-receptor crosstalk. Development *146*, dev178673. https://doi.org/10.1242/dev.178673.

Coppiello, G., Barlabé, P., Moya-Jódar, M., Abizanda, G., Pogontke, C., Barreda, C., Iglesias, E., Linares, J., Arellano-Viera, E., Larequi, E., et al. (2023). Generation of heart and vascular system in rodents by blastocyst complementation. Dev. Cell *58*, 2881–2895.e7. https://doi.org/10.1016/j.devcel.2023.10.008.

Costantini, F., and Kopan, R. (2010). Patterning a complex organ: branching morphogenesis and nephron segmentation in kidney development. Dev. Cell *18*, 698–712. https://doi.org/10.1016/j.devcel.2010.04.008.

Dekel, B., Burakova, T., Ben-Hur, H., Marcus, H., Oren, R., Laufer, J., and Reisner, Y. (1997). Engraftment of human kidney tissue in rat radiation chimera: II. Human fetal kidneys display reduced immunogenicity to adoptively transferred human peripheral blood mononuclear cells and exhibit rapid growth and development. Transplantation *64*, 1550–1558. https://doi.org/10.1097/00007890-199712150-00008.

Dekel, B., Amariglio, N., Kaminski, N., Schwartz, A., Goshen, E., Arditti, F.D., Tsarfaty, I., Passwell, J.H., Reisner, Y., and Rechavi, G. (2002). Engraftment and differentiation of human metanephroi into functional mature nephrons after transplantation into mice is accompanied by a profile of gene expression similar to normal human kidney development. J. Am. Soc. Nephrol. *13*, 977–990. https://doi.org/10.1681/ASN.V134977.

Dekel, B., Burakova, T., Arditti, F.D., Reich-Zeliger, S., Milstein, O., Aviel-Ronen, S., Rechavi, G., Friedman, N., Kaminski, N., Passwell, J.H., and Reisner, Y. (2003). Human and porcine early kidney precursors as a new source for transplantation. Nat. Med. *9*, 53–60. https://doi.org/10.1038/nm812.

Freedman, B.S., Brooks, C.R., Lam, A.Q., Fu, H., Morizane, R., Agrawal, V., Saad, A.F., Li, M.K., Hughes, M.R., Werff, R.V., et al. (2015). Modelling kidney disease with CRISPR-mutant kidney organoids derived from human pluripotent epiblast spheroids. Nat. Commun. *6*, 8715. https://doi.org/10.1038/ncomms9715.

Goto, T., Hara, H., Sanbo, M., Masaki, H., Sato, H., Yamaguchi, T., Hochi, S., Kobayashi, T., Nakauchi, H., and Hirabayashi, M. (2019). Generation of pluripotent stem cell-derived mouse kidneys in Sall1-targeted anephric rats. Nat. Commun. *10*, 451. https://doi.org/10.1038/s41467-019-08394-9.

Griffith, B.P., Goerlich, C.E., Singh, A.K., Rothblatt, M., Lau, C.L., Shah, A., Lorber, M., Grazioli, A., Saharia, K.K., Hong, S.N., et al. (2022). Genetically modified porcine-to-human cardiac xenotransplantation. N. Engl. J. Med. *387*, 35–44. https://doi.org/10.1056/NEJMoa2201422.

Hamanaka, S., Umino, A., Sato, H., Hayama, T., Yanagida, A., Mizuno, N., Kobayashi, T., Kasai, M., Suchy, F.P., Yamazaki, S., et al. (2018). Generation of vascular endothelial cells and hematopoietic cells by blastocyst complementation. Stem Cell Rep. *11*, 988–997. https://doi.org/10.1016/j.stemcr.2018.08.015.

Hatini, V., Huh, S.O., Herzlinger, D., Soares, V.C., and Lai, E. (1996). Essential role of stromal mesenchyme (Foxd1) in kidney morphogenesis. Genes Dev. *10*, 1467–1478. https://doi.org/10.1101/gad.10.12.1467.

Hirata, W., Tomoda, T., Yuri, S., and Isotani, A. (2022). Generation of the Y-chromosome linked red fluorescent protein transgenic mouse model and sexing at the preimplantation stage. Exp. Anim. *71*, 82–89. https://doi.org/10.1538/expanim.21-0119.

Huang, J., He, B., Yang, X., Long, X., Wei, Y., Li, L., Tang, M., Gao, Y., Fang, Y., Ying, W., et al. (2024). Generation of rat forebrain tissues in mice. Cell *187*, 2129–2142.e17. https://doi.org/10.1016/j.cell.2024.03.017.

Isotani, A., Hatayama, H., Kaseda, K., Ikawa, M., and Okabe, M. (2011). Formation of a thymus from rat ES cells in xenogeneic nude mouse↔rat ES chimeras. Genes Cells *16*, 397–405. https://doi.org/10.1111/j.1365-2443.2011.01495.x.

Isotani, A., Yamagata, K., Okabe, M., and Ikawa, M. (2016). Generation of Hprt-disrupted rat through mouse←rat ES chimeras. Sci. Rep. *6*, 24215. https://doi.org/10.1038/srep24215.

James, R.G., Kamei, C.N., Wang, Q., Jiang, R., and Schultheiss, T.M. (2006). Osr1 is required for development of the metanephric kidney and regulates formation and differentiation of kidney precursor cells. Development *133*, 2995–3004. https://doi.org/10.1242/dev.02442.

Kobayashi, A., Valerius, M.T., Mugford, J.W., Carroll, T.J., Self, M., Oliver, G., and McMahon, A.P. (2008). Six2 defines and regulates a multipotent self-renewing nephron progenitor population throughout mammalian kidney development. Cell Stem Cell *3*, 169–181. https://doi.org/10.1016/j.stem.2008.05.020.

Kobayashi, T., Goto, T., Oikawa, M., Sanbo, M., Yoshida, F., Terada, R., Niizeki, N., Kajitani, N., Kazuki, K., Kazuki, Y., et al. (2021). Blastocyst complementation using Prdm14-deficient rats enables efficient germline transmission and generation of functional mouse spermatids in rats. Nat. Commun. *12*, 1328. https://doi.org/10.1038/s41467-021-21557-x.

Kobayashi, T., Yamaguchi, T., Hamanaka, S., Kato-Itoh, M., Yamazaki, Y., Ibata, M., Sato, H., Lee, Y.S., Usui, J.I., Knisely, A.S., et al. (2010). Generation of rat pancreas in mouse by interspecific blastocyst injection of pluripotent stem cells. Cell *142*, 787–799. https://doi.org/10.1016/j.cell.2010.07.039.

Matsunari, H., Watanabe, M., Hasegawa, K., Uchikura, A., Nakano, K., Umeyama, K., Masaki, H., Hamanaka, S., Yamaguchi, T.,

Nagaya, M., et al. (2020). Compensation of disabled organogeneses in genetically modified pig fetuses by blastocyst complementation. Stem Cell Rep. *14*, 21–33. https://doi.org/10.1016/j.stemcr.2019.11.008.

McMahon, A.P. (2016). Development of the mammalian kidney. Curr. Top. Dev. Biol. *117*, 31–64. https://doi.org/10.1016/bs.ctdb.2015.10.010.

Miura, A., Sarmah, H., Tanaka, J., Hwang, Y., Sawada, A., Shimamura, Y., Otoshi, T., Kondo, Y., Fang, Y., Shimizu, D., et al. (2023). Conditional blastocyst complementation of a defective Foxa2 lineage efficiently promotes the generation of the whole lung. eLife *12*, e86105. https://doi.org/10.7554/eLife.86105.

Montgomery, R.A., Stern, J.M., Lonze, B.E., Tatapudi, V.S., Mangiola, M., Wu, M., Weldon, E., Lawson, N., Deterville, C., Dieter, R.A., et al. (2022). Results of two cases of pig-to-human kidney xenotransplantation. N. Engl. J. Med. *386*, 1889–1898. https://doi.org/10.1056/NEJMoa2120238.

Moore, M.W., Klein, R.D., Fariñas, I., Sauer, H., Armanini, M., Phillips, H., Reichardt, L.F., Ryan, A.M., Carver-Moore, K., and Rosenthal, A. (1996). Renal and neuronal abnormalities in mice lacking GDNF. Nature *382*, 76–79. https://doi.org/10.1038/382076a0.

Motohashi, K. (2015). A simple and efficient seamless DNA cloning method using SLiCE from Escherichia coli laboratory strains and its application to SLiP site-directed mutagenesis. BMC Biotechnol. *15*, 47. https://doi.org/10.1186/s12896-015-0162-8.

Mugford, J.W., Sipilä, P., McMahon, J.A., and McMahon, A.P. (2008). Osr1 expression demarcates a multi-potent population of intermediate mesoderm that undergoes progressive restriction to an Osr1-dependent nephron progenitor compartment within the mammalian kidney. Dev. Biol. *324*, 88–98. https://doi.org/10.1016/j.ydbio.2008.09.010.

Naito, Y., Hino, K., Bono, H., and Ui-Tei, K. (2015). CRISPRdirect: software for designing CRISPR/Cas guide RNA with reduced off-target sites. Bioinformatics *31*, 1120–1123. https://doi.org/10.1093/bioinformatics/btu743.

Nishinakamura, R., Matsumoto, Y., Nakao, K., Nakamura, K., Sato, A., Copeland, N.G., Gilbert, D.J., Jenkins, N.A., Scully, S., Lacey, D.L., et al. (2001). Murine homolog of SALL1 is essential for ureteric bud invasion in kidney development. Development *128*, 3105–3115. https://doi.org/10.1242/dev.128.16.3105.

Obara-Ishihara, T., Kuhlman, J., Niswander, L., and Herzlinger, D. (1999). The surface ectoderm is essential for nephric duct formation in intermediate mesoderm. Development *126*, 1103–1108. https://doi.org/10.1242/dev.126.6.1103.

Pascual, M., Theruvath, T., Kawai, T., Tolkoff-Rubin, N., and Cosimi, A.B. (2002). Strategies to improve long-term outcomes after renal transplantation. N. Engl. J. Med. *346*, 580–590. https://doi.org/10.1056/NEJMra011295.

Pichel, J.G., Shen, L., Sheng, H.Z., Granholm, A.C., Drago, J., Grinberg, A., Lee, E.J., Huang, S.P., Saarma, M., Hoffer, B.J., et al. (1996). Defects in enteric innervation and kidney development in mice lacking GDNF. Nature *382*, 73–76. https://doi.org/10.1038/382073a0.

Ran, F.A., Hsu, P.D., Wright, J., Agarwala, V., Scott, D.A., and Zhang, F. (2013). Genome engineering using the CRISPR-Cas9 system. Nat. Protoc. *8*, 2281–2308. https://doi.org/10.1038/nprot.2013.143.

Ran, Q., Zhou, Q., Oda, K., Yasue, A., Abe, M., Ye, X., Li, Y., Sasaoka, T., Sakimura, K., Ajioka, Y., and Saijo, Y. (2020). Generation of thyroid tissues from embryonic stem cells via blastocyst complementation in vivo. Front. Endocrinol. *11*, 609697. https://doi.org/10.3389/fendo.2020.609697.

Riccio, P., Cebrian, C., Zong, H., Hippenmeyer, S., and Costantini, F. (2016). Ret and Etv4 promote directed movements of progenitor cells during renal branching morphogenesis. PLoS Biol. *14*, e1002382. https://doi.org/10.1371/journal.pbio.1002382.

Ruiz-Estevez, M., Crane, A.T., Rodriguez-Villamil, P., Ongaratto, F.L., You, Y., Steevens, A.R., Hill, C., Goldsmith, T., Webster, D.A., Sherry, L., et al. (2021). Liver development is restored by blastocyst complementation of HHEX knockout in mice and pigs. Stem Cell Res. Ther. *12*, 292. https://doi.org/10.1186/s13287-021-02348-z.

Saxén, L. (1987). Organogenesis of the Kidney (Cambridge University Press).

Schuchardt, A., D'Agati, V., Larsson-Blomberg, L., Costantini, F., and Pachnis, V. (1994). Defects in the kidney and enteric nervous system of mice lacking the tyrosine kinase receptor Ret. Nature *367*, 380–383. https://doi.org/10.1038/367380a0.

Schuchardt, A., D'Agati, V., Pachnis, V., and Costantini, F. (1996). Renal agenesis and hypodysplasia in ret-k– mutant mice result from defects in ureteric bud development. Development *122*, 1919–1929. https://doi.org/10.1242/dev.122.6.1919.

Sequeira-Lopez, M.L.S., Lin, E.E., Li, M., Hu, Y., Sigmund, C.D., and Gomez, R.A. (2015). The earliest metanephric arteriolar progenitors and their role in kidney vascular development. Am. J. Physiol. Regul. Integr. Comp. Physiol. *308*, R138–R149. https://doi.org/10.1152/ajpregu.00428.2014.

Shakya, R., Watanabe, T., and Costantini, F. (2005). The role of GDNF/Ret signaling in ureteric bud cell fate and branching morphogenesis. Dev. Cell *8*, 65–74. https://doi.org/10.1016/j.devcel.2004.11.008.

Strell, P., Shetty, A., Steer, C.J., and Low, W.C. (2022). Interspecies chimeric barriers for generating exogenic organs and cells for transplantation. Cell Transplant. *31*, 9636897221110525. https://doi.org/10.1177/09636897221110525.

Sukhatme, V.P. (2003). The Kidney: From Normal Development to Congenital Disease. N. Engl. J. Med. *349*, 2176–2177. https://doi.org/10.1056/NEJM200311273492224.

Taguchi, A., and Nishinakamura, R. (2017). Higher-order kidney organogenesis from pluripotent stem cells. Cell Stem Cell *21*, 730–746.e6. https://doi.org/10.1016/j.stem.2017.10.011.

Takahashi, K., and Yamanaka, S. (2006). Induction of pluripotent stem cells from mouse embryonic and adult fibroblast cultures by defined factors. Cell *126*, 663–676. https://doi.org/10.1016/j.cell.2006.07.024.

Takahashi, K., Tanabe, K., Ohnuki, M., Narita, M., Ichisaka, T., Tomoda, K., and Yamanaka, S. (2007). Induction of pluripotent stem cells from adult human fibroblasts by defined factors. Cell *131*, 861–872. https://doi.org/10.1016/j.cell.2007.11.019.

Takasato, M., Er, P.X., Chiu, H.S., Maier, B., Baillie, G.J., Ferguson, C., Parton, R.G., Wolvetang, E.J., Roost, M.S., Chuva de Sousa

Lopes, S.M., and Little, M.H. (2015). Kidney organoids from human iPS cells contain multiple lineages and model human nephrogenesis. Nature *526*, 564–568. https://doi.org/10.1038/nature15695.

Tanigawa, S., Tanaka, E., Miike, K., Ohmori, T., Inoue, D., Cai, C.-L., Taguchi, A., Kobayashi, A., and Nishinakamura, R. (2022). Generation of the organotypic kidney structure by integrating pluripotent stem cell-derived renal stroma. Nat. Commun. *13*, 611. https://doi.org/10.1038/s41467-022-28226-7.

Throesch, B.T., Bin Imtiaz, M.K., Muñoz-Castañeda, R., Sakurai, M., Hartzell, A.L., James, K.N., Rodriguez, A.R., Martin, G., Lippi, G., Kupriyanov, S., et al. (2024). Functional sensory circuits built from neurons of two species. Cell *187*, 2143–2157.e15. https://doi.org/10.1016/j.cell.2024.03.042.

Usui, J.i., Kobayashi, T., Yamaguchi, T., Knisely, A.S., Nishinakamura, R., and Nakauchi, H. (2012). Generation of kidney from pluripotent stem cells via blastocyst complementation. Am. J. Pathol. *180*, 2417–2426. https://doi.org/10.1016/j.ajpath.2012.03.007.

Wang, J., Xie, W., Li, N., Li, W., Zhang, Z., Fan, N., Ouyang, Z., Zhao, Y., Lai, C., Li, H., et al. (2023). Generation of a humanized mesonephros in pigs from induced pluripotent stem cells via embryo complementation. Cell Stem Cell *30*, 1235–1245.e6. https://doi.org/10.1016/j.stem.2023.08.003.

Wen, B., Li, E., Ustiyan, V., Wang, G., Guo, M., Na, C.L., Kalin, G.T., Galvan, V., Xu, Y., Weaver, T.E., et al. (2021). In vivo generation of lung and thyroid tissues from embryonic stem cells using blastocyst complementation. Am. J. Respir. Crit. Care Med. *203*, 471–483. https://doi.org/10.1164/rccm.201909-1836OC.

Wolfe, R.A., Ashby, V.B., Milford, E.L., Ojo, A.O., Ettenger, R.E., Agodoa, L.Y., Held, P.J., and Port, F.K. (1999). Comparison of mortality in all patients on dialysis, patients on dialysis awaiting transplantation, and recipients of a first cadaveric transplant. N. Engl. J. Med. *341*, 1725–1730. https://doi.org/10.1056/NEJM199912023412303.

Wu, J., Platero-Luengo, A., Sakurai, M., Sugawara, A., Gil, M.A., Yamauchi, T., Suzuki, K., Bogliotti, Y.S., Cuello, C., Morales Valencia, M., et al. (2017). Interspecies chimerism with mammalian pluripotent stem cells. Cell *168*, 473–486.e15. https://doi.org/10.1016/j.cell.2016.12.036.

Yagi, M., Kishigami, S., Tanaka, A., Semi, K., Mizutani, E., Wakayama, S., Wakayama, T., Yamamoto, T., and Yamada, Y. (2017). Derivation of ground-state female ES cells maintaining gamete-derived DNA methylation. Nature *548*, 224–227. https://doi.org/10.1038/nature23286.

Yamaguchi, T., Sato, H., Kato-Itoh, M., Goto, T., Hara, H., Sanbo, M., Mizuno, N., Kobayashi, T., Yanagida, A., Umino, A., et al. (2017). Interspecies organogenesis generates autologous functional islets. Nature *542*, 191–196. https://doi.org/10.1038/nature21070.

Yamaguchi, T., Sato, H., Kobayashi, T., Kato-Itoh, M., Goto, T., Hara, H., Mizuno, N., Yanagida, A., Umino, A., Hamanaka, S., et al. (2018). An interspecies barrier to tetraploid complementation and chimera formation. Sci. Rep. *8*, 15289. https://doi.org/10.1038/s41598-018-33690-7.

Ying, Q.L., Wray, J., Nichols, J., Batlle-Morera, L., Doble, B., Woodgett, J., Cohen, P., and Smith, A. (2008). The ground state of embryonic stem cell self-renewal. Nature *453*, 519–523. https://doi.org/10.1038/nature06968.

Yuri, S., Arisawa, N., Kitamuro, K., and Isotani, A. (2024a). Blastocyst complementation-based rat-derived heart generation reveals cardiac anomaly barriers to interspecies chimera development. iScience *27*, 111414. https://doi.org/10.1016/j.isci.2024.111414.

Yuri, S., Murase, Y., and Isotani, A. (2024b). Generation of rat-derived lung epithelial cells in FGFR2b-deficient mice retains species-specific development. Development *151*, dev202081. https://doi.org/10.1242/dev.202081.

Zvick, J., Tarnowska-Sengül, M., Ghosh, A., Bundschuh, N., Gjonlleshaj, P., Hinte, L.C., Trautmann, C.L., Noé, F., Qabrati, X., Domenig, S.A., et al. (2022). Exclusive generation of rat spermatozoa in sterile mice utilizing blastocyst complementation with pluripotent stem cells. Stem Cell Rep. *17*, 1942–1958. https://doi.org/10.1016/j.stemcr.2022.07.005.

Zhou, Q., Xue, W., Wang, X., Xie, L., Zhang, L., and Zheng, M. (2022). Current status of xenotransplantation research and the immune barriers. Front. Immunol. *13*, 928173. https://doi.org/10.3389/fimmu.2022.928173.

STAR★METHODS

KEY RESOURCES TABLE

REAGENT or RESOURCE	SOURCE	IDENTIFIER
**Antibodies**		
SIX2 Polyclonal antibody (dilution 1:100)	Proteintech	11562-1-AP<br>RRID: AB_2189084
Purified Mouse Anti-E-Cadherin antibody (dilution 1:100)	BD Transduction Laboratories	610181<br>RRID: AB_397581
Pbx1 Antibody (dilution 1:100)	Cell Signaling Technology	4342<br>RRID: AB_2160295
Endomucin (V.7C7)<br>(dilution 1:100)	Santa Cruz Biotechnology	sc-65495<br>RRID: AB_2100037
WT1 (Wilms Tumor Protein) antibody<br>(dilution 1:100)	Abcam	ab89901<br>RRID: AB_2043201
Purified anti-Pax-2 Antibody (dilution 1:100)	Biolegend	901001<br>RRID: AB_2734656
CD31/PECAM-1 Antibody (dilution 1:100)	R and D Systems	AF3628<br>RRID: AB_2161028
Goat Alexa Fluor 647 anti-rabbit IgG (dilution 1:1000)	Thermo Fisher Scientific	A11017<br>RRID: AB_2535814
Goat Alexa Fluor 647 anti-mouse IgG (dilution 1:1000)	Thermo Fisher Scientific	A21237<br>RRID: AB_2535806
Goat Alexa Fluor 647 anti-rat IgG (dilution 1:1000)	Thermo Fisher Scientific	A21247<br>RRID: AB_141778
Donkey Anti-Rabbit IgG H&L (Alexa Fluor® 647)<br>(dilution 1:500-1:1000)	Abcam	ab150075<br>RRID: AB_2752244
Donkey Anti-Mouse IgG H&L (Alexa Fluor® 647)<br>(dilution 1:500-1:1000)	Abcam	ab150107<br>RRID: AB_2535806
Donkey Anti-Goat IgG H&L (Alexa Fluor 647)<br>(dilution 1:500-1:1000)	Abcam	ab150135<br>RRID: AB_2687955
Lotus Tetragonolobus Lectin (LTL), Biotinylated<br>(dilution 1:100)	Vector	B-1325-2
Streptavidin, Alexa Fluor™ 647 Conjugate (dilution 1:500)	Thermo Fisher Scientific	S21374
**Chemicals, peptides, and recombinant proteins**		
CHIR99021	Axon	1386
CGP77675	Sigma	SML0314
PD0325901	Wako	162–25291
N2	Thermo Fisher Scientific	17502048
B27	Thermo Fisher Scientific	17504044
CARD HyperOva	Kyudo	1.0mL
Puromycin	Sigma	P9620

(*Continued on next page*)

Continued

REAGENT or RESOURCE	SOURCE	IDENTIFIER
Hoechst33342	Dojindo	KV072
Opti-MEM	Thermo Fisher Scientific	31985062
2.5% Trypsin	Nacalai Tesque	18172–94
**Critical commercial assays**		
Trizol reagent	Thermo Fisher Scientific	15596026
SuperScript IV VILO master mix	Thermo Fisher Scientific	11756050
GoTaq® Green Master Mix	Promega	M712
KOD Fx *Neo*	TOYOBO	KFX-201
Q5 High-Fidelity DNA polymerase	NEB	M0491S
Luna Universal qPCR Master Mix	NEB	M3003L
Lipofectamine 3000	Thermo Fisher Scientific	L3000008
**Experimental models: Cell lines**		
R01-09 ESC line	Yuri et al. (2024b)	N/A
R01 ESC line	provided from Dr. Ikawa.	N/A
rDby-RFP rat ESC line	This study	N/A
rRFP5-2 rat ESC line	This study	N/A
Sall1-KO ESC lines	This study	N/A
Ret-KO ESC lines	This study	N/A
Ret/Sall1-DKO ESC lines	This study	N/A
Osr1-KO ESC lines	This study	N/A
**Experimental models: Organisms/strains**		
Mouse: *Osr1*-GFP	This study	N/A
Mouse: *Osr1*-Cre	This study	N/A
**Oligonucleotides**		
See Tables S1 and S2	N/A	N/A
**Recombinant DNA**		
pSpCas9(BB)-2A-Puro (pX459) V2.0 plasmids	Addgene	#62988
pLSODN-4D	BioDynamics Laboratory Inc.	DS620
**Software and algorithms**		
CrisperDirect	N/A	https://crispr.dbcls.jp/
ImageJ-Fiji software	NIH images	https://imagej.nih.gov/ij/

EXPERIMENTAL MODEL AND STUDY PARTICIPANT DETAILS

Animals

All animal experiments were conducted in accordance with the guidelines of "Regulations and By-Laws of Animal Experimentation at the Nara Institute for Science and Technology" and were approved by the Animal Experimental Committee at the Nara Institute of Science and Technology (approval no. 2109 and no 0.2103) and NCGG Animal Ethics Committee (approval no. 7–13). Heterozygous of *Osr1*-GFP or *Osr1*-Cre mouse line were established from chimeras that *Osr1*-GFP ESCs or *Osr1*-Cre ESCs derived from R01 ESCs were injected into ICR embryo at E2.5 stage. After establishment of *Osr1*-GFP or *Osr1*-Cre mouse lines, B6D2F1 mice were used for backcrossing. B6D2F1 and ICR mice were purchased from Japan SLC, Inc.

Cell culture

For the establishment of *Sall1*-KO ESC lines, two different gRNAs were designed to delete the entire exon region of *Sall1* gene. The two plasmids were then transfected into R01-09 ESCs, which used in previous study (Yuri et al., 2024a, 2024b), using Lipofectamine 3000 (Thermo Fisher Scientific). To establish *Ret*-KO ESC lines, a DNA fragment which contain EGFP-polyA sequence was inserted into the *Ret* gene. The EGFP sequence was inserted in-frame within the *Ret* gene. To enhance homologous recombination, a gRNA was designed close to knock-in site of the *Ret* gene. Two different plasmids—the targeting vector and the plasmid inducing a double-strand break in genomic DNA—were then transfected into R01-09 ESCs. For *Osr1*-GFP or *Osr1*-Cre ESC lines, the EGFP-polyA or hCre-polyA sequence was inserted into the *Osr1* gene. The EGFP or hCre sequence was inserted in-frame within the *Osr1* locus. Similarly to *Ret*-KO ESCs, a gRNA was designed close to knock-in site of the *Osr1* gene. Two different plasmids were then transfected into R01 ESCs. Transfected cells were cultured with transient treatment using 1 μg/mL puromycin (Sigma) for 2 days and then passaged for clonal culture. ESC colonies were subjected to genotyping by PCR and sequencing. ESCs were cultured on gelatin- and MEF (mouse embryonic fibroblast)-coated dish in N2 (Gibco) and B27 medium (Gibco) supplemented with 3 μM CHIR99021 (AXON), 1.5 μM CGP77675 (SIGMA), and mouse LIF (NPO in Osaka University) (N2B27-a2i/L medium). *Osr1*-KO ESC lines were established from blastocysts obtained by crossing *Osr1*-GFP and *Osr1*-Cre mouse line and were cultured in N2B27-a2i/L medium (Yagi et al., 2017; Choi et al., 2017).

A rat ESC line, rDby-RFP, was established from blastocysts obtained by crossing DA female with rGBGS#6 (F344) male rats (Isotani et al., 2016) and was cultured with N2B27 medium supplemented with 3 μM CHIR99021, 1.5 μM PD0325901 (Wako), mouse LIF and human LIF (Sigma) (N2B27-2i/L medium). A CAG-tdTomato-T2A-puroR cassette was subsequently inserted into genomic DNA between introns 1 and 2 of the *Ddx3y* locus. Another rat ESC line, rRFP5-2, was similarly derived from blastocysts obtained by crossing DA females with rGBGS#6 (F344) males. The pCAG-tdTomato-T2A-puroR plasmid was transfected into the established male ESCs, followed by puromycin selection for seven days, resulting in a stable ESC line with constitutive tdTomato expression. These rat ESCs were cultured on gelatin (Sigma)- or matrigel (Corning)- and MEF-coated dishes in N2B27-2i/L medium (Ying et al., 2008). These cell lines were cultured under sterile conditions and tested negative for mycoplasma contamination. The cell source is provided in key resources table.

METHOD DETAILS

Plasmid construction

The oligo DNAs for the target gRNA sequence of *Sall1*, *Ret*, and *Osr1* genes (Table S7) were inserted into BbsI site of the pSpCas9(BB)-2A-Puro (pX459) V2.0 plasmid, which was a gift from Feng Zhang through Addgene (plasmid # 62988) (Ran et al., 2013). The target sites were designed using the CrisperDirect website to identify specific target sites (Naito et al., 2015). For the Ret-GFP plasmid, left-arm and MluI site + right-arm PCR fragments were inserted into the EcoRV site of the pLSODN-4D plasmid (BioDynamics Laboratory Inc.) using the SLiCE method (Motohashi et al., 2015). The EGFP-polyA fragment was then inserted into the MluI site using the SLiCE method. For the Osr1-GFP plasmid, the left-arm, EGFP-polyA, and right-arm fragments were simultaneously inserted into the EcoRV site of the pLSODN-4D plasmid using SLiCE method. For the Osr1-Cre plasmid, left-arm, hCre-polyA, and right-arm fragments were simultaneously inserted into the EcoRV site of pLSODN-4D plasmid using the SLiCE method. EGFP-polyA or hCre-polyA fragment was amplified from pCX-EGFP or pCX-hCre provided from Dr. Masahito Ikawa (Osaka University), respectively.

For the rDby-RFP plasmid, the 3′-arm PCR fragment was inserted into the SpeI site of pCAG-tdTomato-2A-PuroR (Hirata et al., 2022) using the SLiCE method, followed by insertion of the 5′-arm PCR fragment into the HindIII site. The oligo

DNAs for the target gRNA sequence of rat *Ddx3y* gene (Table S7) were inserted into BbsI site of the pSpCas9(BB)-2A-Puro (pX459) V2.0 plasmids. PCR fragments were amplified with Q5 High-Fidelity DNA polymerase (New England Biolabs) or KOD FX *Neo* (Toyobo). Primer information for plasmid construction is stated in Table S7.

Genotyping

Primers for detecting *Sall1*-KO, *Ret*-KO, *Osr1*-KO and rDby-RFP are listed in Table S7. Primers for detecting *Osr1*-GFP and *Osr1*-Cre mice are also shown in Table S7. DNA fragments were amplified using GoTaq (Promega) for 40 cycles to detect null or WT alleles under the following conditions: 94°C for 30 s, 60°C for 30 s and 72°C for 30–90 s. DNA fragments for *Sall1*-KO detection were amplified using KOD FxNeo (Toyobo).

ESCs injection

ICR, *Osr1*-GFP or *Osr1*-Cre female mice aged at 8–12 weeks were treated with CARD HyperOva and hCG for superovulation and then mated with ICR or *Osr1*-GFP or *Osr1*-Cre male mice, respectively. Two-cell-stage embryos were collected from the oviduct of female mice 42–46 h after hCG injection using the flush-out method. The collected embryos were incubated in KSOM medium at 37°C under 5% CO_2 conditions until injection was performed. The rBC method was performed as described in previous reports (Yuri et al., 2024a, 2024b). Briefly, 6–8 cells of *Sall1*-KO, *Ret*-KO or *Osr1*-KO ESCs were injected into ICR embryos at 8-cell-stage. For the intraspecies BC method with mouse embryos, 6–8 cells of R01-09 ESCs were injected into embryos obtained from intercrosses between *Osr1*-GFP and *Osr1*-Cre mice at the E3.5 stage. For interspecies BC method, 4 cells of rDby-RFP or rRFP5-2 ESCs were injected into embryos obtained from matings between *Osr1*-GFP and *Osr1*-Cre mice at the E3.5 stage. The injected embryos were transferred into the uteri of E2.5 pseudopregnant ICR mice. For rat ESC injection into rat embryos, Wistar-Imamichi males and females were naturally mated to obtain blastocysts. The blastocysts were then injected by 4–6 rDby-RFP ESCs and transferred into the uteri of E3.5 pregnant Wistar-Imamichi rats. Embryos were dissected at E13.5 or E14.5 for mouse experiments and at E15.5 for the rat experiments. Chimeras were analyzed based on RFP or GFP signal under a fluorescent stereomicroscope (Leica; MZFL III).

Flow cytometry analysis and fluorescence-assisted cell sorting

For mouse–mouse ESCs or mouse–rat ESCs chimera experiments, embryos were recovered at the E14.5 or E13.5 stage. For rat–rat ESCs chimera experiments, embryos were recovered at the E15.5 stage. Tail, lung, or kidney samples were incubated with 0.25% trypsin/EDTA for 10 min at 37°C. After pipetting to dissociate the tissues, 10% FBS in PBS was added, and the cell suspensions were filtered through a 37-μm mesh. The FL3 detector on an Accuri flow cytometer (BD Bioscience) or the ECD detector on a CytoFLEX S flow cytometer (Beckman coulter) was used to detect RFP+ populations. An MA900 cell sorter (SONY) was used to isolate RFP+ and RFP- subpopulations for RT-PCR analysis.

RNA expression analysis

Total RNA was purified using TRIzol reagent (Thermo Fisher Scientific). cDNA was synthesized using the SuperScript IV VILO master mix (Thermo Fisher Scientific). For quantitative RT-PCR analysis, Luna Universal qPCR Master Mix (New England Biolabs) was used for amplification, and amplified products were detected using a LightCycler 96 (Roche). The species specificity of all primer sets was evaluated based on the amplification and melting curves obtained from qPCR results. Primers used for RT-PCR are listed in Table S8.

Immunocytochemistry staining

The kidneys at E14.5 were fixed with 4% paraformaldehyde (PFA) in phosphate buffered saline (PBS) (−) (Nacalai) for overnight at 4°C. After washing with PBS (−), the tissues were immersed in 10, 15, and 20% sucrose in PBS (−). The treated tissues were then sunk into Tissue-TeK O.C.T compound (Sakura Finetek). After making sections with a cryostat (Leica; NX70) at 10 μm thickness, the slides were dried at 25°C, followed by washing with PBS (−). In the mouse–rat chimera experiments, the kidneys were cultured on 0.4 μm isopore polycarbonate filter (Millipore) with DMEM supplemented with 10% FBS for 5–7 days and then fixed and analyzed as same protocol as described above. For Immunostaining method were previously described (Yuri et al., 2024a, 2024b). The antibodies used in this study were listed in key resources table. Immunostained slides were observed using a laser confocal microscope (LSM710, LSM900; Zeiss, Mica; Leica).

QUANTIFICATION AND STATISTICAL ANALYSIS

Statistical analysis

For quantification of immunostaining, different nonoverlapped regions were analyzed in each marker. Statistical analyses were performed by unpaired Student's *t* test with significance at *: $p < 0.05$, **: $p < 0.01$. Error bars represent mean ± standard deviation (SD). For quantitative RT-PCR data expressed as relative fold changes, all values are expressed as mean ± standard deviation from at least triplicate experiments. Student's *t* test for unpaired comparisons was performed and results at $p < 0.01$ were considered statistically significant.

Stem Cell Reports, Volume *21*

Supplemental Information

Rat cell-derived kidney generation via interspecies blastocyst complementation in an *Osr1*-KO mouse model

Shunsuke Yuri and Ayako Isotani

Fig.S1

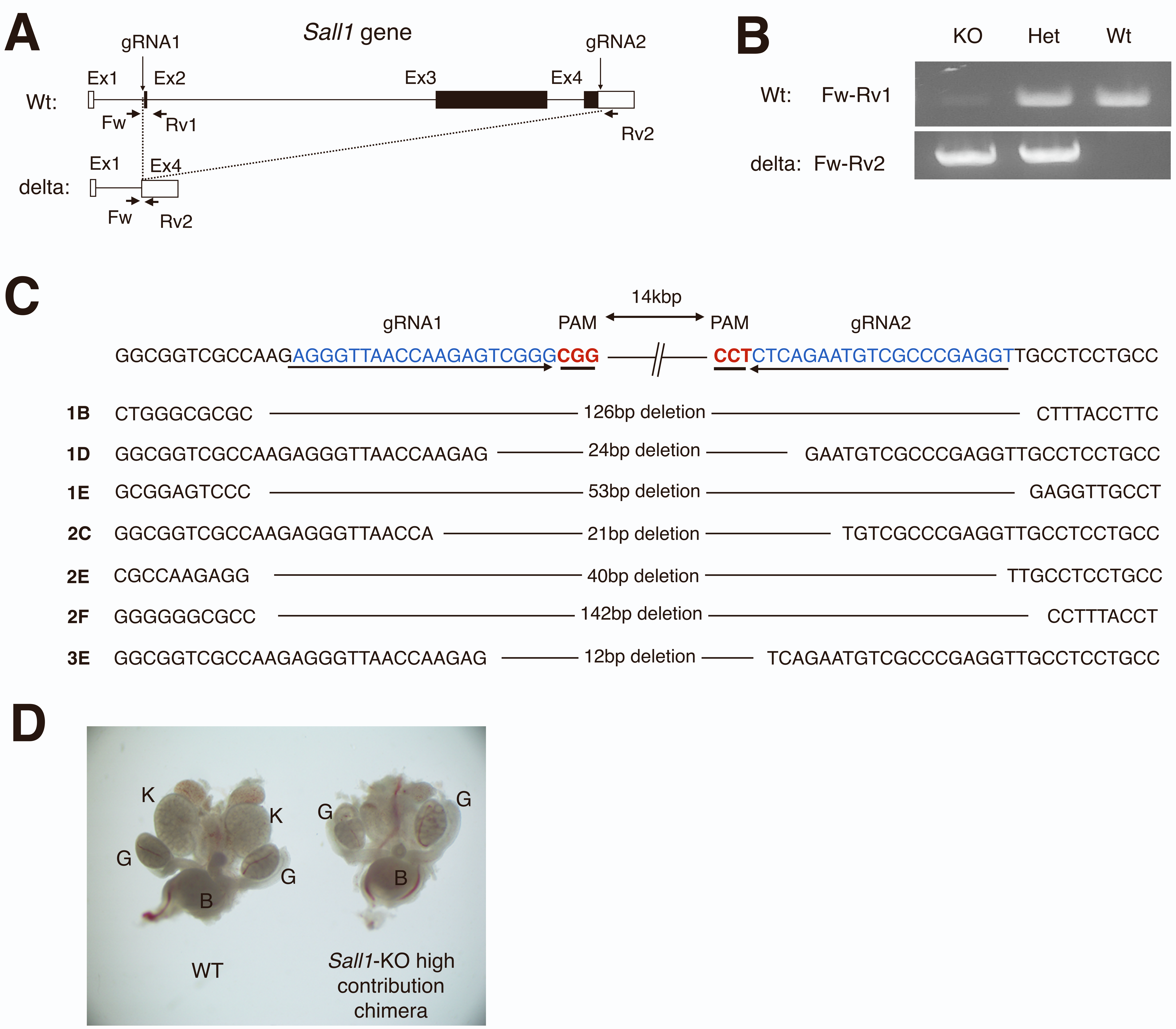

Figure S1. Generation of *Sall1*-KO ESC lines, related to Figure 1.
(A) Strategy for generating the *Sall1* knockout (KO) model. Both gRNA1 and gRNA2 were used to delete exons 1–4 of the *Sall1* gene.
(B) Genotypes of *Sall1*-KO, *Sall1* heterozygous (Het), and *Sall1* WT ESCs.
(C) Mutation patterns of the obtained *Sall1*-KO ESC lines.
(D) Representative images of the urogenital region in WT and *Sall1*-KO chimeras with high *Sall1*-KO cell contribution.

Fig.S2

A

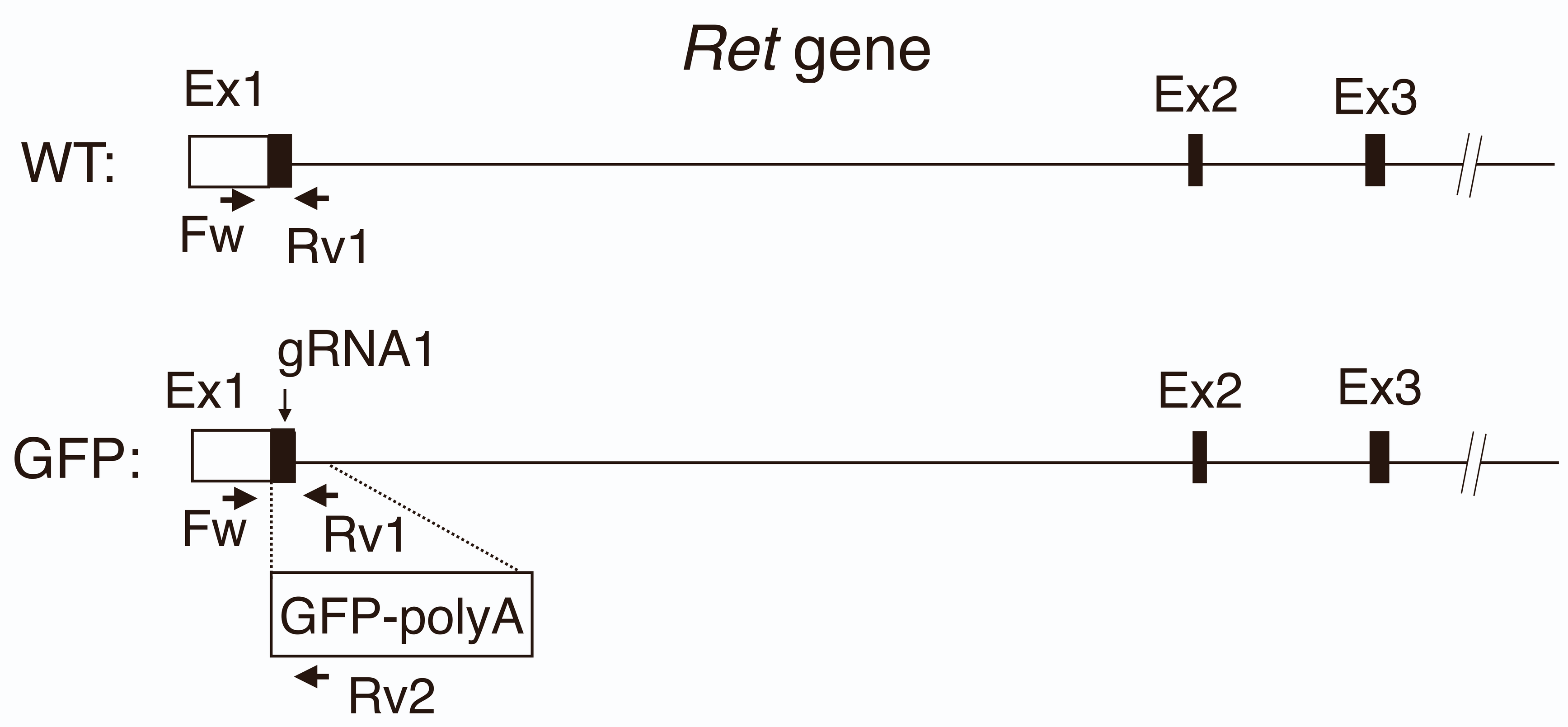

B

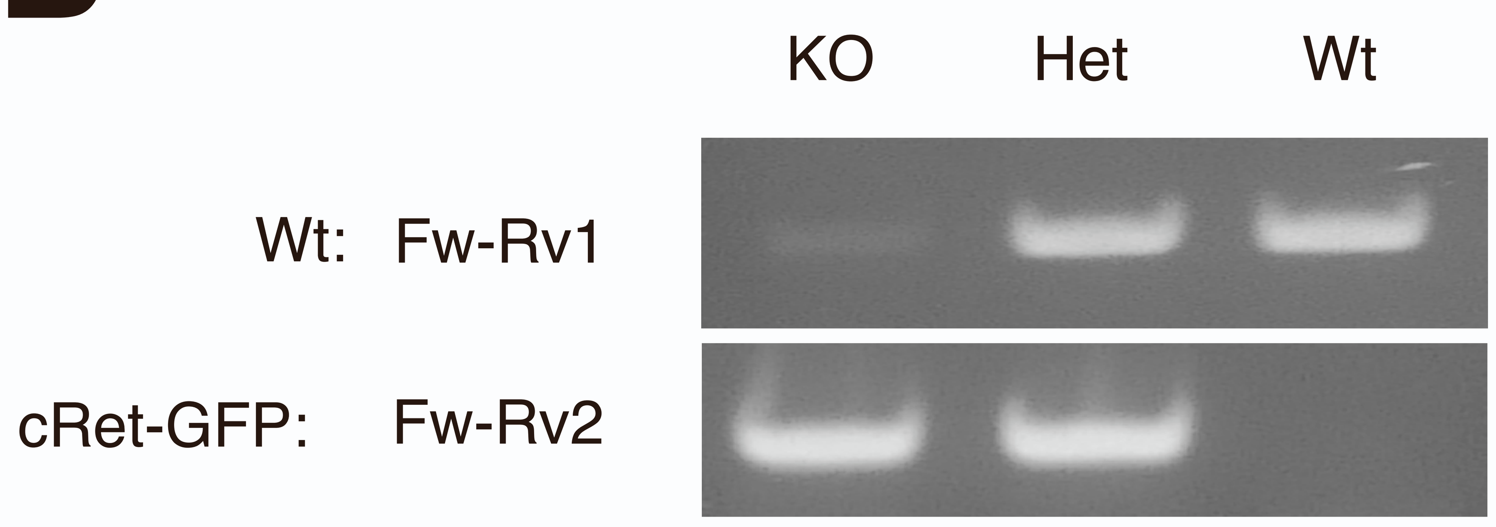

C

clone	allele 1	allele 2
**7B**	GFP insertion	1 bp addition (Frame shift)
**7E**	GFP insertion	1 bp addition (Frame shift)
**9D**	GFP insertion	GFP insertion

D

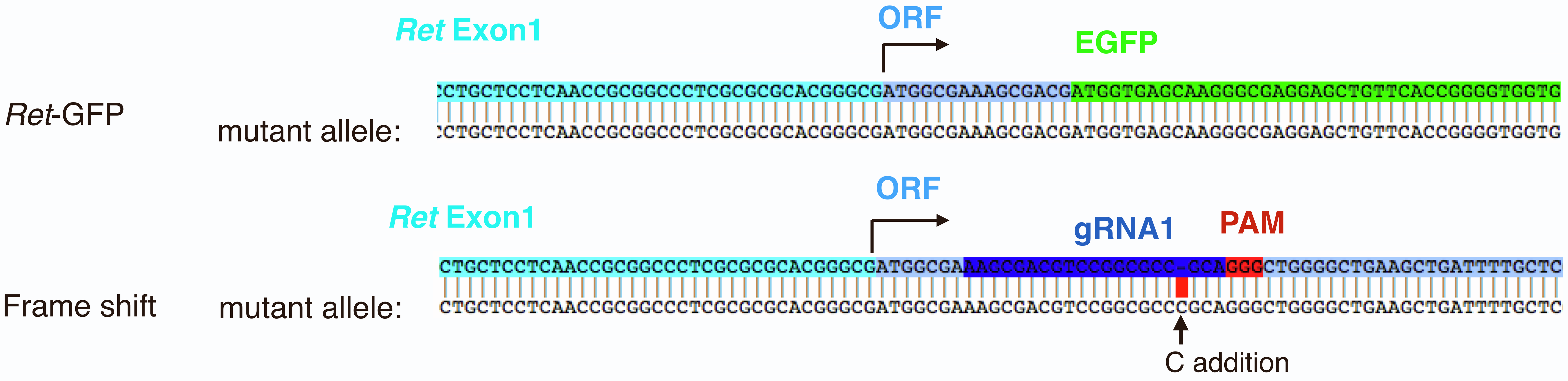

E

clone	*Ret* allele 1	*Ret* allele 2
**1A**	GFP insertion	GFP insertion
**1H**	GFP insertion	GFP insertion
**5D**	GFP insertion	GFP insertion

Figure S2. Generation of *Ret*-KO ESC lines, related to Figure 2.

(A) Strategy for generating the *Ret* knockout (KO) model. A GFP–polyA cassette was inserted in-frame within the *Ret* gene.

(B) Genotypes of *Ret*-KO, *Ret* heterozygous (Het), and *Ret* WT ESCs.

(C–D) Mutation patterns of the obtained *Ret*-KO ESC lines.

(E) Mutation patterns of the obtained *Ret*/*Sall1*-KO ESC lines.

Fig.S3

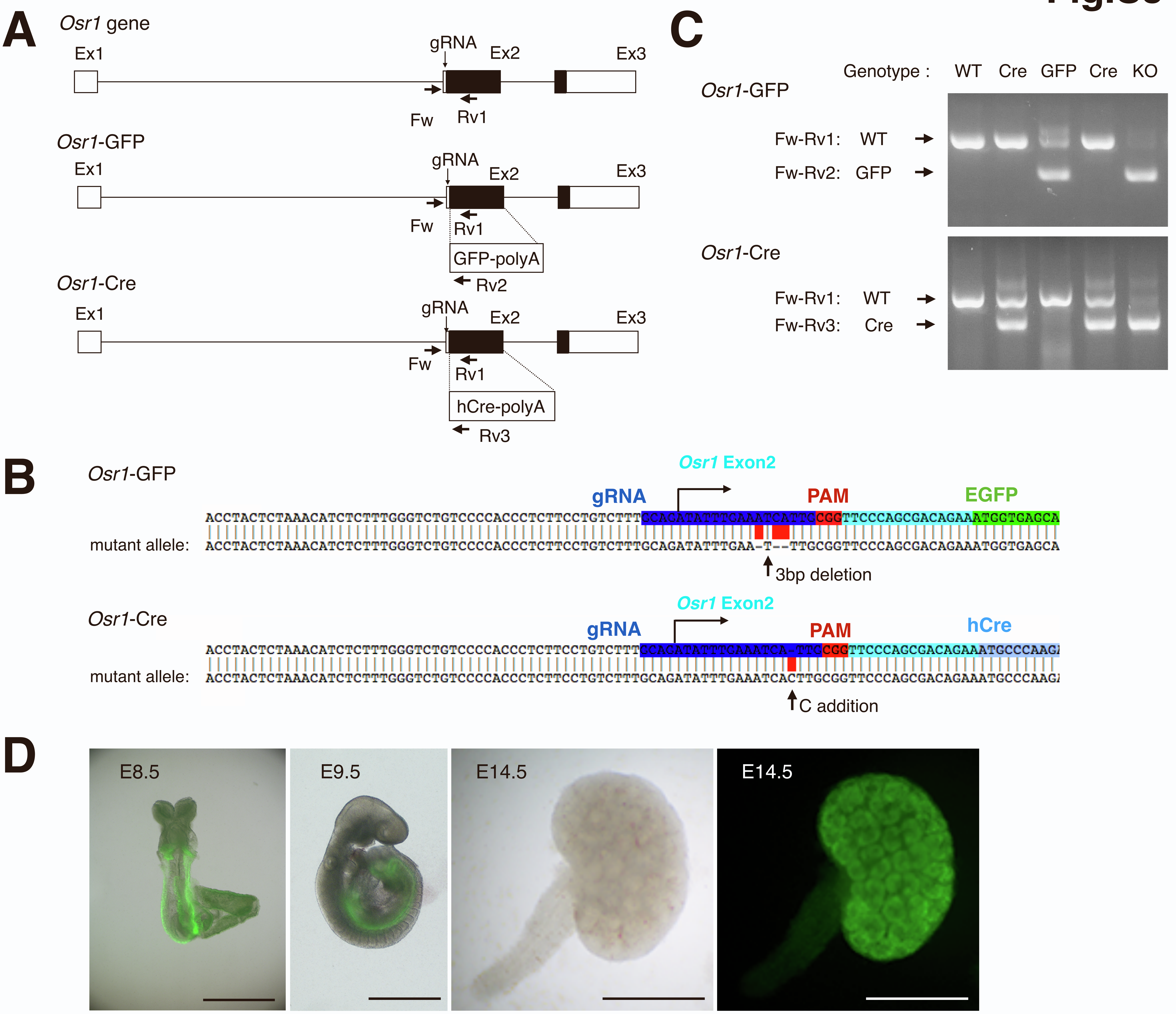

Figure S3. Generation of *Osr1*-GFP and *Osr1*-Cre ESC lines, related to Figure 3 and 4.
(A) Strategy for generating the *Osr1*-GFP and *Osr1*-Cre ESC lines. A GFP–polyA cassette or an hCre–polyA cassette was inserted in-frame within the *Osr1* gene.
(B) Mutation patterns of the obtained *Osr1*-GFP and *Osr1*-Cre ESC lines.
(C) Genotypes of the embryos obtained from *Osr1*-GFP and *Osr1*-Cre mouse lines.
(D) Representative images of GFP expression patterns in *Osr1*-GFP embryos at E8.5 and E9.5, and in the E14.5 kidney. Scale bars: 1 mm (E8.5 and E9.5), 500 µm (E14.5 kidney).

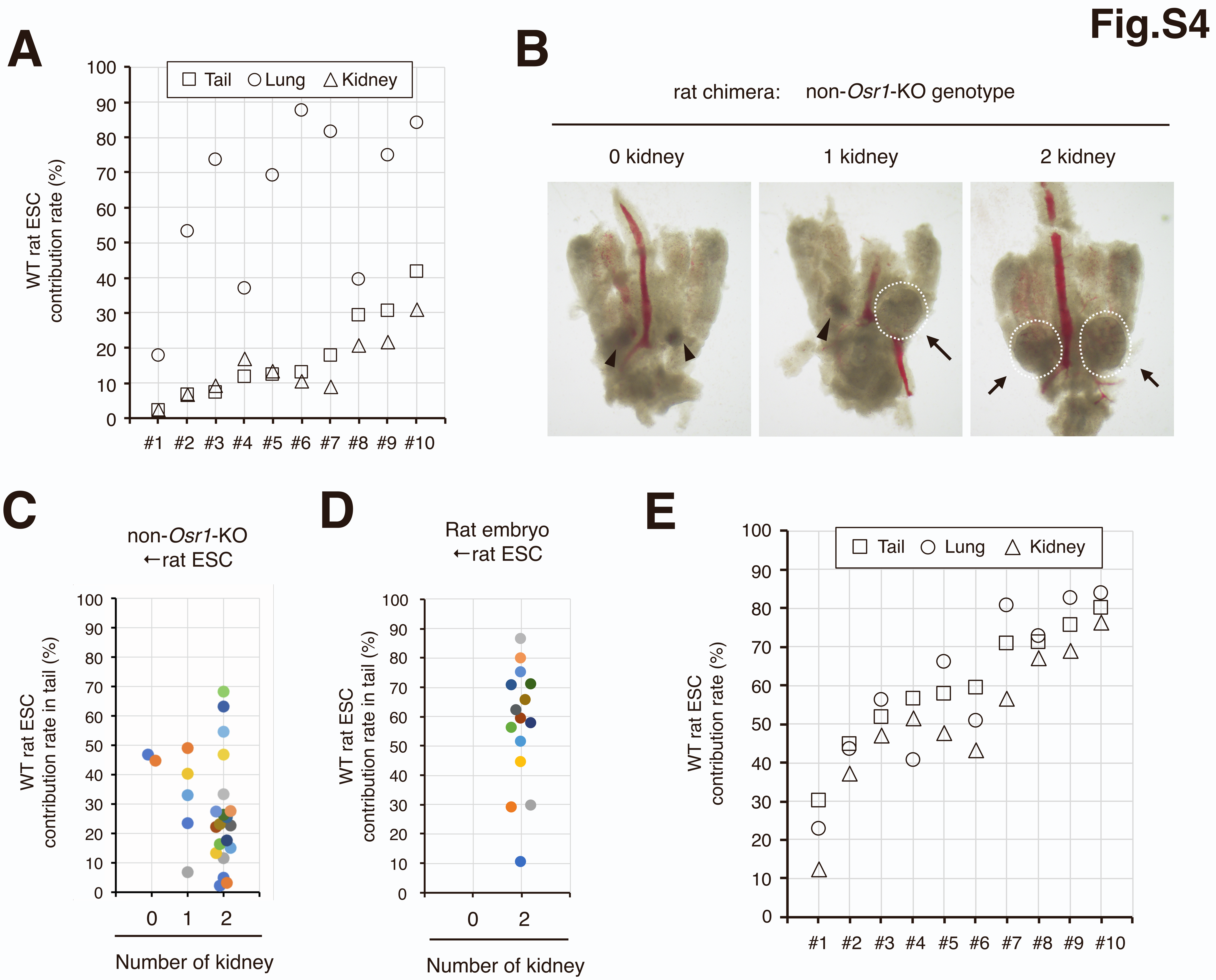

Figure S4. Characterization of kidneys in the rat ESC chimeras, related to Figure 5.

(A) Representative cellular contribution rates of the injected rat WT cells in the tail, lung, and kidney of chimeras with a non-*Osr1*-KO genotype.

(B) Representative urogenital images obtained from chimeras with a non-*Osr1*-KO genotype. The black arrow and white dot circle indicate a kidney, and the black arrowhead indicates a vestigial kidney structure.

(C) Relationship between the cellular contribution rate of donor (rat) cells in the tail and the presence of kidneys in non-*Osr1*-KO-rat chimeras. Non-*Osr1*-KO–rat chimeras without kidneys (n=2), with one kidney (n=5), and with two kidneys (n = 20) were analyzed.

(D) Relationship between the cellular contribution rate of donor (rat) cells in the tail and the presence of kidneys in rat-rat chimeras. WT rat–rat chimeras with two kidneys (n = 15) were analyzed.

(E) Representative cellular contribution rates of the injected rat WT cells in the tail, lung, and kidney of rat–rat chimeras.

Fig.S5

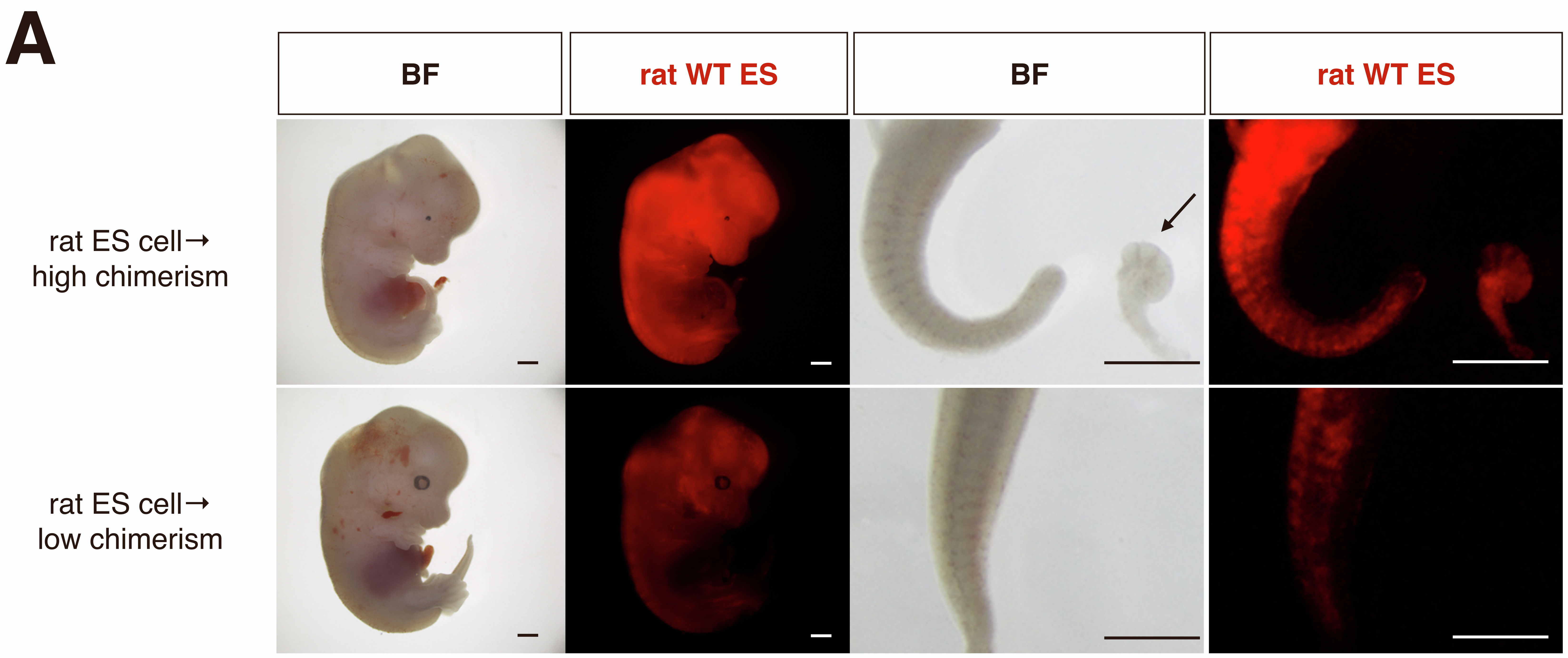

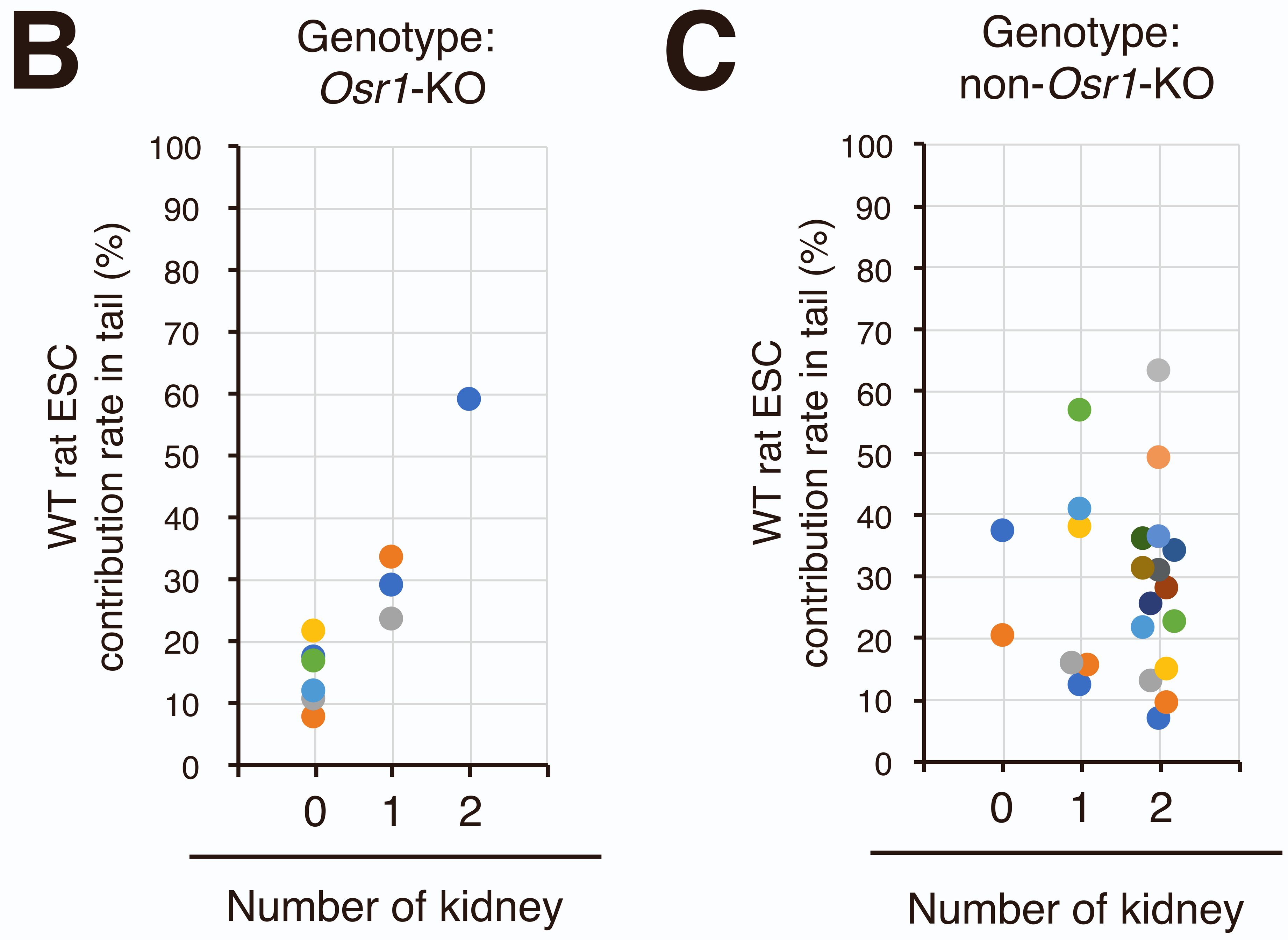

D

Genotype	WT	Het	KO
Chimera	11	25	10
non-Chimera	12	32	8

Figure S5. Interspecies BC method for generating rat kidney with *Osr1*-KO mouse model, related to Figure 5.

(A) Representative images of embryos and kidneys derived from chimeras generated from mouse *Osr1*-KO embryos and rat ESCs (RFP+: rRFP5-2), showing high rat contribution (upper) and low rat contribution (lower). Chimeras with high rat contribution contained kidneys (black arrow) in the *Osr1*-KO background, and RFP-expressing kidneys were observed. Scale bars, 1 mm.

(B) Relationship between the cellular contribution rate of injected rat WT cells in the tail and the presence of kidneys in *Osr1*-KO and WT rat ESC chimeras. *Osr1*-KO-rat WT ESC (rRFP5-2) chimeras without kidneys (n = 6), one kidney (n = 3), or two kidneys (n = 1) were analyzed.

(C) Relationship between the cellular contribution rate of donor (rat) cells in the tail and the presence of kidneys in non-*Osr1*-KO ESC chimeras. Non-*Osr1*-KO-rat ESC (rRFP5-2) chimeras without kidneys (n = 2), with one kidney (n = 6), or with two kidneys (n = 15) were analyzed.

(D) Genotype results of interspecies blastocyst complementation using rRFP5-2.

Fig.S6

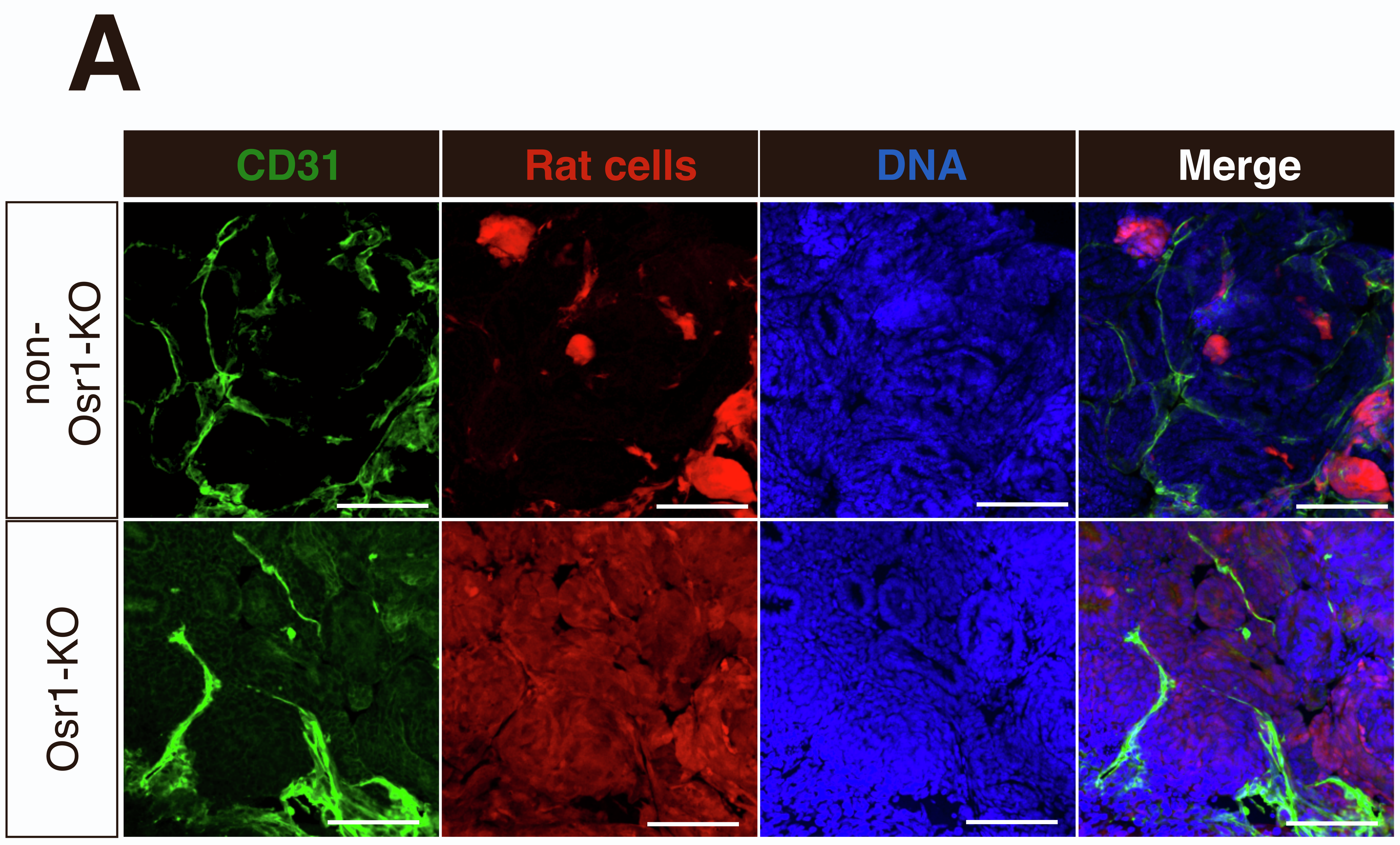

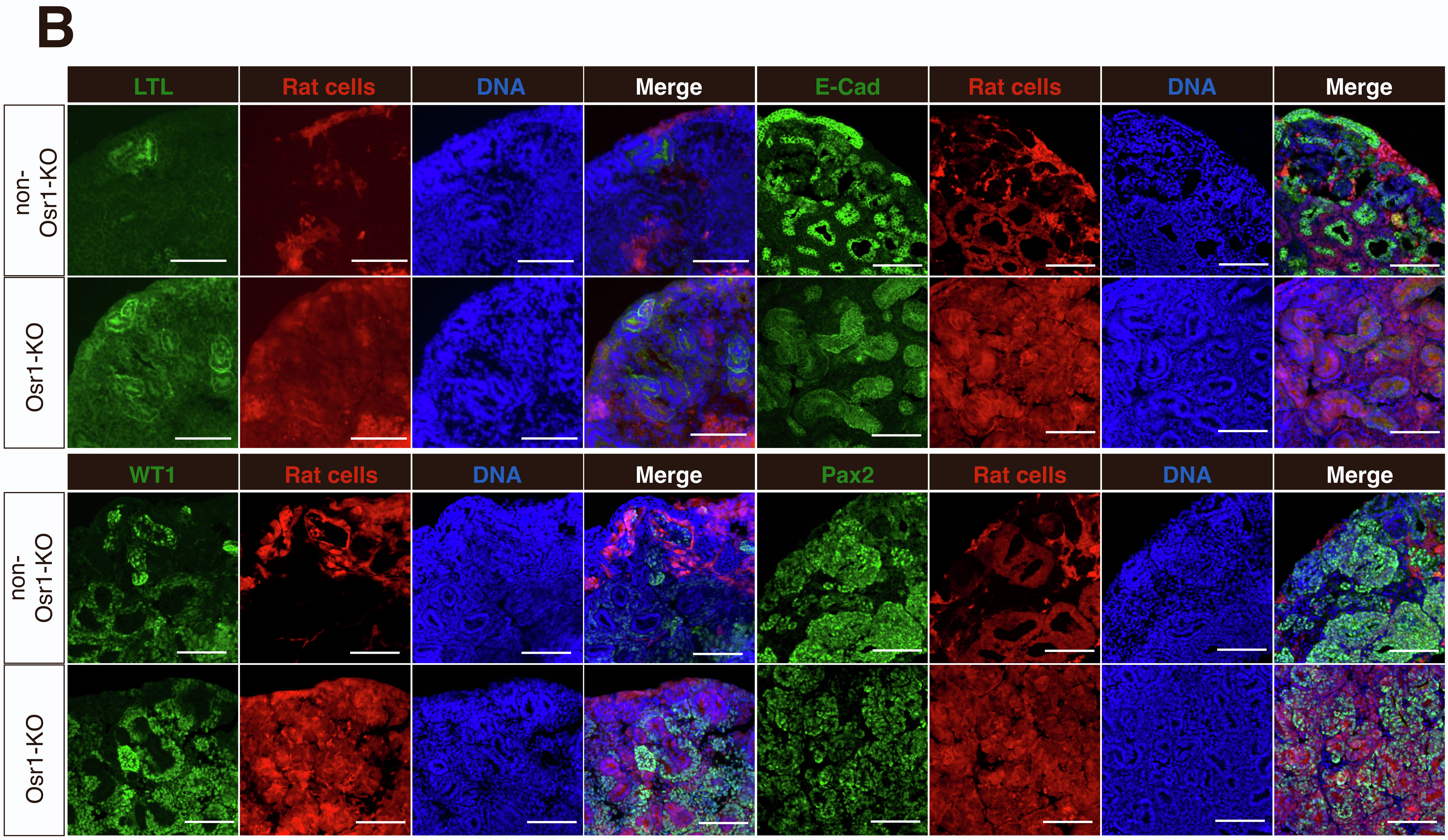

Figure S6. Immunofluorescence analysis of kidneys in the interspecies chimeras, related to Figure 5.

(A) Representative immunostaining image of CD31 in kidney of *Osr1*-KO and rat WT cell chimera or non-*Osr1*-KO and rat WT cell chimera. The kidney tissues were organ-cultured for 5-7 days. Scale bars, 100 µm.

(B) Representative immunostaining image of LTL, E-Cad, WT1 and Pax2 in kidney of *Osr1*-KO and rat WT cell chimera or non-*Osr1*-KO and rat WT cell chimera. The kidney tissues were organ-cultured for 5-7 days. Scale bars, 100 µm.

Table S1. Results of *Sall1*-KO ESC injection with the rBC method (donor: mouse ESCs; host: mouse embryo), related to Figure 1

ESC line	Transplantation	Implantation	Live embryos	RFP+ chimera
Sall1 -KO ESCs (1B)	31	19	10	11
Sall1 -KO ESCs (1D)	31	22	6	3
Sall1 -KO ESCs (1E)	30	23	13	8
Sall1 -KO ESCs (2C)	30	22	11	6
Sall1 -KO ESCs (2E)	30	22	13	9
Sall1 -KO ESCs (2F)	30	23	10	7
Sall1 -KO ESCs (3E)	71	40	20	14
WT ESCs (R01-09)	129	85	48	37

Table S2. Results of *Ret*-KO and *Ret/Sall1*-DKO ESC injection with the rBC method (donor: mouse ESCs; host: mouse embryo), related to Figure 2

ESC line	Transplantation	Implantation	Live embryos	RFP+ chimera
Ret -KO ESCs (7B)	40	33	16	7
Ret -KO ESCs (7E)	183	116	81	59
Ret -KO ESCs (9D)	20	18	8	3
Ret/Sall1 -DKO ESCs (1A)	63	49	18	9
Ret/Sall1 -DKO ESCs (1H)	30	22	6	4
Ret/Sall1 -DKO ESCs (5D)	107	67	45	36

Table S3. Results of *Osr1*-KO ESC and WT ESC injection with the rBC method (donor: mouse ESCs; host: mouse embryo), related to Figure 3

ESC line	Transplantation	Implantation	Live embryos	RFP+ chimera
Osr1 -KO ESCs (#4)	15	9	7	7
Osr1 -KO ESCs (#6)	16	9	5	2
Osr1 -KO ESCs (#9)	18	8	7	6
Osr1 -KO ESCs (#12)	45	31	18	13
Osr1 -KO ESCs (#15)	48	29	22	14
Osr1 -KO ESCs (#23)	46	31	27	15
Osr1 -KO ESCs (#24)	42	22	18	11
WT ESCs (#3-2)	20	15	13	12

Table S4. Results of mouse WT ESC injection with the intraspecies BC method (donor: mouse ESCs; host: mouse embryo), related to Figure 4

ESC line	Transplantation	implantation	live embryos	RFP+ chimera
WT mouse ESCs	868	514	318	153

Table S5. Results of rat WT ESC injection with the interspecies BC method (donor: rat ESCs; host: mouse embryo), related to Figure 5 and Figure S5

ESC line	Transplantation	implantation	live embryos	RFP+ chimera
rDby-RFP	724	385	164	60
rRFP-5-2	817	443	98	46

Table S6. Results of rat WT ESC injection in rat embryos (donor: rat ESCs; host: rat embryo), related to Figure 5 and Figure S4

ESC line	Transplantation	RFP+ chimera
rDby-RFP	76	23

Table S7. Oligonucleotide information, related to STAR Methods

ID	oligo name	sequence	comment
#1	Sall1-sgRNA1-F	caccAGGGTTAACCAAGAGTCGGG	
#2	Sall1-sgRNA1-R	aaacCCCGACTCTTGGTTAACCCT	
#3	Sall1-sgRNA2-F	caccACCTCGGGCGACATTCTGAG	
#4	Sall1-sgRNA2-R	aaacCTCAGAATGTCGCCCGAGGT	
#5	Sall1-KO check F	ATTAAGGCGAGCGGGTTTGA	
#6	Sall1-KO check R	AGAGGCATTTGCTCTTGTAAGG	#5 & #6: Sall1-KO allele check
#7	Sall1-WT check R	GAAAGCTCCAGCCGGGATAG	#5 & #7: Sall1-WT allele check
#8	Ret-sgRNA1-F	caccAAGCGACGTCCGGCGCCGCA	
#9	Ret-sgRNA1-R	aaacTGCGGCGCCGGACGTCGCTT	
#10	pLSODN-4D+Ret-Left-F	atgcctgcaggctcttcgatATCCTTCCCAACACCCCTGTAG	
#11	Ret-Left-R	CGTCGCTTTCGCCATCG	#10 & #11: left-arm amplification for Ret-GFP
#12	MluI+Ret Right arm F	ggcgatggcgaaagcgacgacgcgtAGTAGACTGCTCCGCCAAAG	
#13	pLSODN-4D+Ret-Right-R	gtggcaatgcccgggatgatCACCTTAGGACCCAGGAACA	#12 & #13: right-arm amplification for Ret-GFP
#14	Ret-EGFP-F	ggcgatggcgaaagcgacgATGGTGAGCAAGGGCGAG	
#15	Ret-EGFP-R	ctttggcggagcagtctactGTCGAGGGATCTTCATAAGAGAAGAG	#14 & #15: EGFP+polyA amplification for Ret-GFP
#16	cRet left check F1	GGGAAGGGAGAGTTCGATTC	
#17	GFP Rv seq primer 1	AACTTGTGGCCGTTTACGTC	#16 & #17: left-arm knock-in check for Ret-GFP
#18	cRet Right check R1	GGAGGGAAGTTGCTCAACAC	
#19	PolyA Fw seq primer 1	CACTCGGAAGGACATATGGG	#18 & #19: right-arm knock-in check for Ret-GFP
#20	cRet Genome check F1	CCGGTGACAGCCAATGTAAG	
#21	cRet Genome check R1	CCGGTGACAGCCAATGTAAG	#20 & #21: WT allele check for Ret-GFP
#22	pLSODN-4D+Osr1-Left-F	atgcctgcaggctcttcgatTGTGAAAGAGCCCTACCGC	
#23	GFP+Osr1-Left-R	tcctcgcccttgctcaccatTTCTGTCGCTGGGAACCG	#22 & #23: left-arm amplification for Osr1-GFP
#24	Osr1-Left+GFP-F	tgcggttcccagcgacagaaATGGTGAGCAAGGGCGAG	
#25	Osr1-Right+GFP-R	gcagtgctgcagtgtcgcacGTCGAGGGATCTTCATAAGAGAAGAG	#24 & #25: EGFP+polyA amplification for Osr1-GFP
#26	GFP+Osr1-Right-F	tcttatgaagatccctcgacGTGCGACACTGCAGCACTG	
#27	pLSODN-4D+Osr1-Right-R	gtggcaatgcccgggatgatTGAGTTGAGTACCGCCTTTTGG	#26 & #27: right-arm amplification for Osr1-GFP
#28	hCre+Osr1-Left-R	ttcctcttcttcttgggcatTTCTGTCGCTGGGAACCG	#22 & #28: left-arm amplification for Osr1-Cre
#29	polyA+Osr1-Right-F	gatccctcgacctgcagcccGTGCGACACTGCAGCACTG	#27 & #29: right-arm amplification for Osr1-Cre
#30	hCre-F	ATGCCCAAGAAGAAGAGGAAGGTG	
#31	polyA-R	GGGCTGCAGGTCGAGGGATC	#30 & #31: hCre+polyA amplification for Osr1-Cre
#32	Check Osr1 Left F	AATTTCCCATGCATCCTGAG	#17 & #32: left-arm knock-in check for Osr1-GFP
#33	hCre check R1	AGCATCTTCCAGGTGTGCTC	#17 & #33: left-arm knock-in check for Osr1-Cre
#34	Check Osr1 Right R	CGGAGTTTTCGTTGTGTGTG	#19 & #33: right-arm knock-in check for Osr1-GFP and Osr1-Cre
#35	Osr1 genome check R1	GCGAGGCTTGGTCTTAAGTG	#17 & #35 & #36 : genotype for Osr1-GFP
#36	Osr1 genome check F1	TATGTTGAGGGGGCAGTAGG	#33 & #35 & #36 : genotype for Osr1-Cre
#37	rDdx3y 5'-Fw2	accatgattacgccaagctCCTTGACCAGCAAGTGAGTTGG	
#38	rDdx3y 5'-Rev1	tcgacctgcagcccaagctCAGTGGTAGTGCAGGCACAC	#37 & #38: 5'-arm amplification for rDby-RFP
#39	rDdx3y 3'-Fw1	gattactattaataactagtcaataatcaatgtcCTTGACCTTCCCACCTCCCC	
#40	rDdx3y 3'-Rev1	gacattgattattgactagAAGCTCTTCTGATGTCCAAGGGTC	#39 & #40: 3'-arm amplification for rDby-RFP
#41	rDdx3y _sgRNA-F	caccGCACTACCACTGAATCCGCT	
#42	rDdx3y_sgRNA-R	aaacAGCGGATTCAGTGGTAGTGC	

Table S8. Primer information for RT-PCR analysis, related to STAR Methods.

ID	oligo name	sequence
#1	RT-mGapdh F	CATTTGCAGTGGCAAAGTGGAG
#2	RT-mGapdhR	CGTCAGATCCACGACGGAC
#3	RT-mrSix2 F	TGTGGCTCAAGGCGCACTAC
#4	RT-mSix2 R	ACGGGTAGGGGTTGTGAGCG
#5	RT-rSix2 R	CGACGGGTAAGGGTTGTGC
#6	RT-mRet F	AGAGTGAGTTACGAGACCTGCTG
#7	RT-mRet R	CAATCTTGCGGCTGTCACGG
#8	RT-rRet F	GGATCCACATCGATGCGGGC
#9	RT-rRet R	GCACAGCCTGGCCAATGACAC
#10	RT-mFoxd1 F	TCAAGATCCCGCGCGAACCG
#11	RT-mFoxd1 R	CCGCGTGGGGAGCGAGTAG
#12	RT-rFoxd1 F	CGACCGCCGGTGCACACGC
#13	RT-rFoxd1 R	CAGCCGCTTGCACGGCGCAG
#14	RT-mouse Pecam F	TGACTTCCAGACTCTCGAGG
#15	RT-mouse Pecam R	TACTCGACAGGATGGAAATCAC
